# Incensole Acetate Regulates the Sirtuin–Insulin Signaling Axis in an Experimental Alzheimer’s Disease Model

**DOI:** 10.3390/ph19101573

**Published:** 2026-10-03

**Authors:** Rabia Kalkan Cakmak, Bahar Sarikamis Johnson, Nilufer Ercin, Nail Besli, Merve Beker, Ulkan Celik

**Affiliations:** 1 Department of Medical Biology, Hamidiye School of Medicine, University of Health Sciences, Istanbul 34668, Turkey; 2 Department of Medical Biology, Hamidiye Institute of Health Sciences, University of Health Sciences, Istanbul 34668, Turkey; 3 Department of Medical Biology, School of Medicine, Atlas University, Istanbul 34408, Turkey; 4 Department of Medical Biology, Hamidiye International School of Medicine, University of Health Sciences, Istanbul 34668, Turkey

**Keywords:** Alzheimer’s disease, incensole acetate, insulin signaling, sirtuins, stem cell

## Abstract

**Background/Objectives:** Alzheimer’s disease (AD) is associated with impaired brain insulin signaling, oxidative stress, and neuroinflammation. This study investigated the effects of incensole acetate (IA) on sirtuin-associated insulin-signaling and cellular stress-response pathways using integrated computational and in vitro approaches. **Methods:** ADMET prediction, molecular docking, molecular dynamics simulations, and MM-PBSA analyses were performed for IA against SIRT1, SIRT3, and SIRT6 and compared with their corresponding reference ligands. An Aβ1–42-induced AD model was established in mesenchymal stem cell-derived neurons. The effects of IA on insulin-signaling components, sirtuins, Aβ, NF-κB, NRF2, and PPARα were evaluated using RT-qPCR, Western blotting, and immunofluorescence. **Results:** IA showed weaker docking affinities than the corresponding reference ligands for all three sirtuins, whereas trajectory-based analyses revealed target-dependent dynamic behavior. MM-PBSA-derived binding-energy estimates were −35.16 ± 3.32, −30.24 ± 3.63, and −38.26 ± 3.90 kcal/mol for IA with SIRT1, SIRT3, and SIRT6, respectively, with IA showing a more favorable relative energetic profile than 8L9 for SIRT6. In vitro, IA treatment was associated with reduced detectable Aβ levels and modulation of INSR, IRS, AKT, and AS160 expression. IA increased SIRT1 and SIRT6 expression, whereas SIRT3 protein levels were not significantly altered. IA also reduced NF-κB and increased NRF2 and PPARα expression. **Conclusions:** IA modulated insulin-signaling-related, sirtuin-associated, inflammatory, oxidative-stress, and metabolic markers in an Aβ1–42-induced cellular AD model. Computational analyses support potential IA–sirtuin interactions, particularly a favorable relative MM-PBSA energetic profile for SIRT6; however, direct sirtuin activation or causal sirtuin-mediated signaling was not established.

## 1. Introduction

Alzheimer’s disease (AD) is a multifactorial, progressive neurodegenerative disorder characterized by memory loss, cognitive dysfunction, and a decline in problem-solving and decision-making abilities. Major pathobiological symptoms are extracellular amyloid plaques and intracellular neurofibrillary tangles formed by hyperphosphorylated tau protein [1,2]. AD accounts for approximately 70–80% of all dementia cases worldwide, which causes a plethoric burden to caregivers and the health system. Existing therapies predominantly alleviate symptoms rather than offer a cure [3].

Besides being an anabolic hormone, insulin has various roles in cell growth and differentiation [4]. Upon binding to the insulin receptor (IR, INSR) on the cell surface, IR undergoes autophosphorylation, subsequently activating the mitogen activated protein kinase (MAPK) and phosphatidylinositol 3-kinase (PI3K)-AKT pathways. Activation of the MAPK pathway induces a mitogenic response and triggers cell proliferation, growth, survival and differentiation. Meanwhile, activation of the PI3K/AKT pathway regulates GLUT4 translocation through AS160; gluconeogenesis through FOXO; glycogen synthesis through GSK3; apoptosis; cell cycle; protein synthesis; lipogenesis and lipolysis via SREBP [5,6].

In the central nervous system, insulin, insulin like growth factor 1 and 2 (IGF1 and IGF2), insulin receptor and insulin like growth factor receptor (IGFR) expressions are concentrated in the hippocampus, hypothalamus and cortex. Especially, insulin resistance in the hippocampus contributes to cognitive impairment and neuronal dysfunction [7,8]. At the molecular level, insensitivity results from decreased receptor levels, reduced receptor affinity for insulin, or impairments in the insulin signaling pathway. These cause disruption of neuroplasticity, neurotransmitter release, metabolism regulation and cognitive functions [9].

Approximately 29% of type 2 diabetes mellitus (T2DM) patients develop cognitive dysfunction and neurodegeneration later in life. T2DM represents the most prevalent comorbidity associated with AD. T2DM medications cause improvement of neurological symptoms caused by dementia [8,10,11]. Patients with AD have insulin function impairment in their peripheric tissues and hyperglycemia. Hyperglycemia affects brain functions negatively via glucose neurotoxicity, vascular injury and advanced glycation end products (AGEs). Peripheral hyperinsulinemia causes a decrease in the amount of insulin receptors in the blood–brain barrier and therefore the insulin level in the brain. Furthermore, it is observed that insulin receptors did not localize in the cell membrane and receptor affinity decreased in AD patients when compared to the control group [12].

Peripheral insulin resistance aggravates amyloid beta accumulation [12,13]. In addition, amyloid beta binds to the insulin receptor, inhibiting its phosphorylation, and therefore disturbs the insulin signaling cascade [7,14]. Hyperinsulinemia resulting from insulin resistance further impairs amyloid beta clearance by the insulin degrading enzyme (IDE) [11].

Due to the decrease in PI3K/AKT cascade signaling caused by brain insulin resistance, the decline in GSK3 inhibitory phosphorylation causes tau hyperphosphorylation. Moreover, excess insulin inhibits protein phosphatase 2 (PP2A), which normally dephosphorylates tau [15].

Glucose hypometabolism in the brain characterized by reduced glucose utilization is one of the fundamental causes of synaptic dysfunction and synapse loss [11].

Considering these findings, AD has been increasingly referred to as “type 3 diabetes” or “brain diabetes,” reflecting the substantial overlap between AD pathology and impaired cerebral insulin signaling [16].

SIRT1, which has functions in energy homeostasis, oxidative stress and apoptosis, also has regulatory roles in insulin secretion, beta cell protection and adipose biogenesis. Decreased SIRT1 levels have been detected in the serum and brain tissues of patients with T2DM and AD [17,18,19]. SIRT3 localized in the mitochondria modulates mitochondrial protein activity through protein deacetylation. A decrement in SIRT3 levels causes insulin resistance, and its level is decreased in AD patients’ brain tissue [17,20]. SIRT6 is localized in the nucleus and has functions in energy metabolism, inflammation, glucose homeostasis and normal functioning of beta cells. In AD patients’ serum, SIRT6 levels were decreased in parallel to SIRT1 and SIRT3 decrement [17,21].

Trees in the Boswellia genus have been used in traditional medicine since 4000 BC in various civilizations, such as Persian, Egyptian and Babylonians, with different purposes like respiratory and digestive system diseases treatment and in religious rites. Boswellia trees contain bioactive compounds, including 11-Keto-β-Boswellic acid (KBA), Acetyl-11-keto-β-Boswellic Acid, incensole and incensole acetate [22,23,24,25]. In rat models of AD, Boswellia extract has been shown to ameliorate behavioral impairment and inhibit acetylcholinesterase. Meanwhile, incensole acetate showed an anti-inflammatory effect by inhibiting NF-κB, reducing oxidative stress, and preventing apoptosis in various experimental models [25,26,27].

In this study, mesenchymal stem cells (MSCs) were differentiated into neurons, and an in vitro Alzheimer’s disease model was established by applying the amyloid beta 1–42 peptide. In silico analyses were performed to investigate the potential interactions of incensole acetate with SIRT1, SIRT3 and SIRT6. Molecular docking was used to characterize predicted ligand–protein interactions, while molecular dynamics simulations and MM-PBSA analyses were employed to evaluate the dynamic behavior and interaction energetics of the complexes. For the in vitro part, neuronal differentiation was shown by immunofluorescent staining with neuronal markers. Next, the treatment dose of incensole acetate was determined by MTT analysis. The effects of IA on insulin-signaling-related components were subsequently evaluated at the transcript and/or protein levels, as appropriate. SIRT1, SIRT3, SIRT6, NF-κB, and NRF2 expressions were determined by Western blot, while PPARα expression was examined by immunofluorescent staining.

## 2. Results

### 2.1. Drug-likeness and ADMET Prediction

Physicochemical properties, absorption, distribution, metabolism, excretion, and toxicity (ADMET) properties of the reference activator molecules 4TQ for SIRT1, BBI for SIRT3 and 8L9 for SIRT6 and incensole acetate were assessed to evaluate the drug likeness properties of incensole acetate and are depicted in Table 1.

Among all molecules, incensole acetate demonstrated prominent physicochemical properties, with optimal molecular weight, low topological surface area and zero Lipinski violations. However, BBI and 8L9 had two and one violations respectively, which indicate poor gastrointestinal absorptions. Regarding BBB penetrations, only IA was estimated as BBB permeant, which is a significant requirement for candidate molecules for neurodegenerative disease treatment.

Consistent with their hydrophobic natures, all reference molecules were predicted as poorly soluble, while IA was moderately soluble.

Regarding cytochrome P450 (CYP) inhibition, IA was predicted to inhibit CYP2C9 and CYP3A4, whereas 4TQ was predicted to inhibit CYP2C19, CYP2C9, CYP2D6, and CYP3A4, and 8L9 was predicted to inhibit CYP2C9. In the toxicity predictions, IA showed H-HT and DILI probabilities of 0.729 and 0.766, respectively. The corresponding values for the positive-control compound AFB1 were higher, reaching 0.881 for H-HT and 0.995 for DILI. IA showed a comparatively lower predicted carcinogenicity probability (0.458) and rat oral acute toxicity probability (0.276), whereas AFB1 showed markedly higher values of 0.987 and 0.886, respectively. These findings indicate that, although IA displayed a more favorable toxicity profile than the positive control, its predicted hepatotoxicity, DILI potential, and CYP2C9/CYP3A4 inhibition represent safety signals requiring further experimental investigation. For toxicity classifications, IA and BBI showed moderate hepatotoxicity potential, and 8L9 had the highest Drug Induced Liver Injury score. According to toxicophore rules, no acute toxicity prediction was determined for any ligand.

### 2.2. Molecular Docking Analysis of Incensole Acetate–Sirtuin Complexes

**Figure 1:**
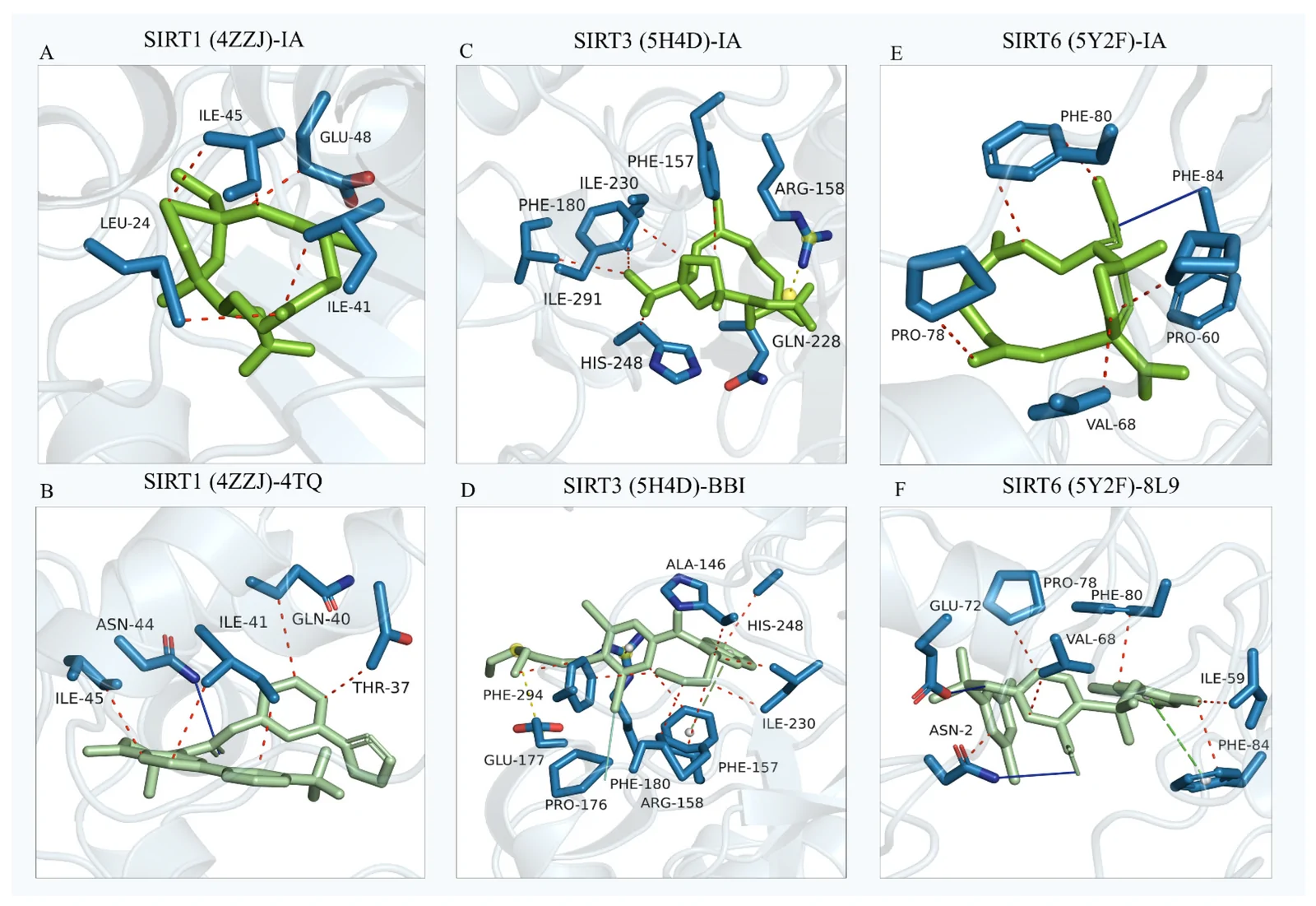
Predicted binding modes and molecular interactions of IA (**A**) and 4TQ (**B**) with SIRT1; IA (**C**) and BBI (**D**) with SIRT3; and IA (**E**) and 8L9 (**F**) with SIRT6 (Protein residues are shown in blue, while ligands are shown in green. Dashed lines and solid lines representing hydrophobic interactions and hydrogen bonds respectively).

To elucidate the predicted interactions of incensole acetate with SIRT1, SIRT3 and SIRT6 proteins and to compare its predicted binding efficiency with reference activator molecules, molecular docking analysis was conducted, and the results are depicted in Table 2 and Figure 1.

IA showed weaker predicted docking affinities than the corresponding reference ligands for all three sirtuins. The binding affinities of IA were −3.270, −5.944, and −5.244 kcal/mol toward SIRT1, SIRT3, and SIRT6, respectively, compared with −6.246 kcal/mol for 4TQ, −6.960 kcal/mol for BBI, and −7.572 kcal/mol for 8L9. Among the three IA–sirtuin complexes, IA exhibited its most favorable docking score toward SIRT3 (−5.944 kcal/mol).

In the 4ZZJ (SIRT1) conformation, IA formed hydrophobic bonds with LEU24, ILE41, ILE45, and GLU48 (3.36–3.66 Å) and no hydrogen bonds, while 4TQ hydrogen bonded with ASN44 and employed hydrophobic interactions with THR37, GLN40, ILE41, ILE45.

Against the 5H4D (SIRT3) conformation, IA had an H-bond with ARG158, same position with reference activator BBI, suggesting a reasonable fit to the active site of SIRT3 protein. IA made hydrophobic interactions (3.39–4.18 Å) with PHE157, PHE180, ILE230, and HIS248, which were common with BBI, while GLN228 and ILE291 were specific in IA; ALA146, ARG158, PRO176, GLU177 and PHE 294 were observed only in BBI.

In the case of 5Y2F (SIRT6), IA had an H-bond with PHE84, while 8L9 H-bonded with ASN2 and GLU72. IA made hydrophobic interactions with VAL68, PRO78, and PHE80 that were common with 8L9, while PRO60 was specific in IA, and ASN2, ILE59 and PHE84 were observed only in 8L9.

As a positive-control assessment of the docking protocol, the selective SIRT1 inhibitor EX-527 was docked into the experimentally characterized inhibitor-binding pocket of SIRT1 (PDB: 4I5I). The top-ranked pose exhibited a predicted binding affinity of −10.672 kcal/mol and was stabilized predominantly by aromatic and hydrophobic interactions involving PHE273, PHE297, ILE316, ILE347, ILE411, and PHE413. Additional van der Waals contacts were observed with ALA262, SER265, ILE270, ILE279, GLN345, ASN346, HIS363, and VAL412. One unfavorable donor–donor interaction with ASP348 was also detected (Supplementary Figure S1).

### 2.3. Molecular Dynamics Simulations Results

In this study, molecular dynamics simulations of docking complexes of reference activator molecule-protein and incensole acetate-protein were conducted. The root mean square deviation (RMSD) and root mean square fluctuation (RMSF) values were determined to analyze the structural integrity and stability of the complexes.

**Figure 2:**
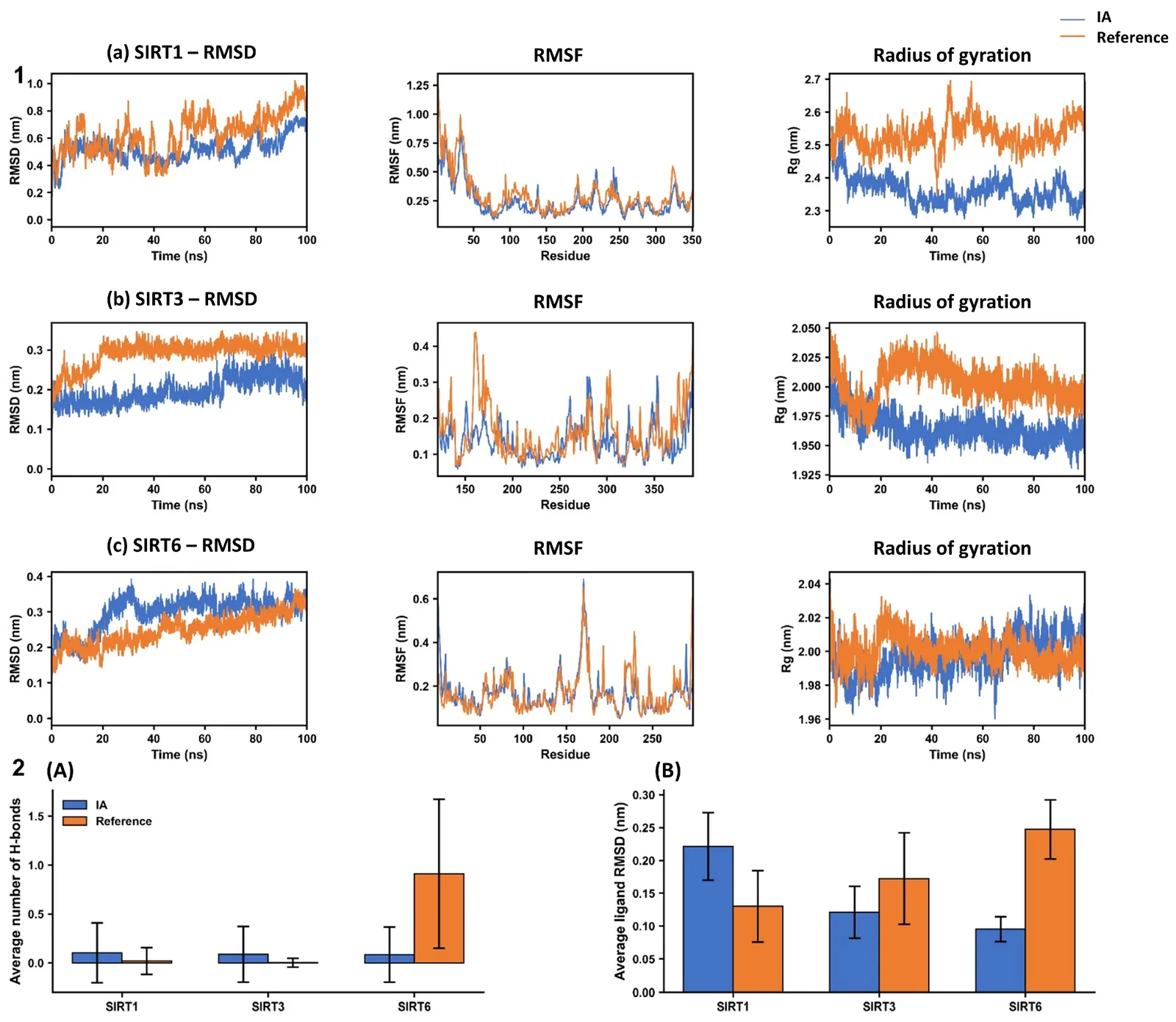
(**1**) Molecular dynamics simulation analyses of SIRT1 (**a**), SIRT3 (**b**) and SIRT6 (**c**) in complex with incensole acetate (IA; blue curves) and reference activator molecules (orange curves; 4TQ for SIRT1, BBI for SIRT3 and 8L9 for SIRT6). For each target, panels show (from left to right) root-mean-square deviation (RMSD), root-mean-square fluctuation (RMSF) and radius of gyration (Rg). (**2**) Average protein–ligand hydrogen bonds (**A**) and average ligand RMSD (**B**) over the 100 ns trajectories (mean ± SD). In (**A**,**B**), blue bars denote IA complexes, and orange bars denote reference complexes.

The temporal evolution of backbone RMSD revealed distinct stabilization patterns among the investigated complexes (Figure 2a–c). For SIRT1 (Figure 2a), the IA complex displayed a relatively stable intermediate interval during the middle portion of the trajectory, followed by an increase in RMSD toward the later stages of the simulation. The SIRT1–4TQ complex showed a more pronounced time-dependent upward drift, particularly during the second half of the trajectory. Therefore, neither SIRT1 complex exhibited a single persistent equilibrium plateau throughout the complete 100 ns simulation.

For SIRT3 (Figure 2b), the BBI complex underwent an initial structural adjustment during approximately the first 20 ns and subsequently maintained a relatively stable RMSD plateau around 0.30–0.31 nm. The SIRT3–IA complex exhibited lower overall RMSD values but showed a more gradual conformational adjustment, followed by a comparatively stable late-stage interval.

For SIRT6 (Figure 2c), the IA complex showed an initial increase in RMSD during approximately the first 20 ns, after which it remained relatively stable around 0.31–0.32 nm for the remainder of the simulation. In contrast, the SIRT6–8L9 complex exhibited a gradual increase in RMSD throughout the trajectory and did not show a clearly persistent plateau. These observations indicate target- and ligand-dependent stabilization behavior and do not support assignment of a single equilibration time across all six systems.

Across the complete 100 ns trajectories, the mean RMSD values were 0.628 and 0.518 nm for SIRT1–4TQ and SIRT1–IA, 0.291 and 0.196 nm for SIRT3–BBI and SIRT3–IA, and 0.248 and 0.297 nm for SIRT6–8L9 and SIRT6–IA, respectively.

Residue-level flexibility was further evaluated by RMSF analysis (Figure 2a–c). For SIRT1, the mean RMSF values were 0.309 nm for the 4TQ complex and 0.252 nm for the IA complex. For SIRT3, the corresponding mean RMSF values were 0.161 nm for BBI and 0.139 nm for IA. In contrast, the SIRT6 complexes exhibited highly similar mean RMSF values of 0.166 nm for 8L9 and 0.170 nm for IA. Although localized fluctuation peaks were observed in individual regions of each protein, the overall RMSF profiles indicated broadly comparable residue-level flexibility between IA-bound and reference-ligand-bound sirtuin complexes, with lower mean fluctuations for IA in SIRT1 and SIRT3 and nearly identical values for the two SIRT6 complexes.

Radius of gyration (Rg) was evaluated to monitor changes in the overall compactness of the protein structures throughout the simulations (Figure 2a–c). For SIRT1, the Rg ranges were 2.351–2.696 nm for the 4TQ complex and 2.273–2.556 nm for the IA complex. For SIRT3, the corresponding ranges were 1.959–2.049 nm for BBI and 1.930–2.017 nm for IA, whereas for SIRT6, they were 1.967–2.035 nm for 8L9 and 1.960–2.043 nm for IA. Overall, the Rg profiles showed relatively limited variations, with no indication of major global expansion or collapse of the investigated protein structures during the 100 ns trajectories.

Protein–ligand hydrogen-bonding patterns were further compared using the average number of hydrogen bonds throughout the simulations (Figure 2A). The mean numbers of protein–ligand hydrogen bonds were 0.10 ± 0.31 for SIRT1–IA and 0.02 ± 0.14 for SIRT1–4TQ, 0.09 ± 0.29 for SIRT3–IA and 0.002 ± 0.04 for SIRT3–BBI, and 0.09 ± 0.28 for SIRT6–IA and 0.91 ± 0.76 for SIRT6–8L9. Among these complexes, SIRT6–8L9 exhibited the highest average number of protein–ligand hydrogen bonds.

Ligand RMSD was additionally evaluated to compare the positional behavior of IA and the corresponding reference ligands during the simulations (Figure 2B). The mean ligand RMSD values were 0.22 ± 0.05 nm for SIRT1–IA and 0.13 ± 0.05 nm for SIRT1–4TQ, 0.12 ± 0.04 nm for SIRT3–IA and 0.17 ± 0.07 nm for SIRT3–BBI, and 0.10 ± 0.02 nm for SIRT6–IA and 0.25 ± 0.05 nm for SIRT6–8L9. IA therefore exhibited lower ligand RMSD than the corresponding reference ligand in the SIRT3 and SIRT6 complexes, whereas 4TQ exhibited a lower ligand RMSD than IA in the SIRT1 complex.

### 2.4. MM-PBSA Analysis Results

**Figure 3:**
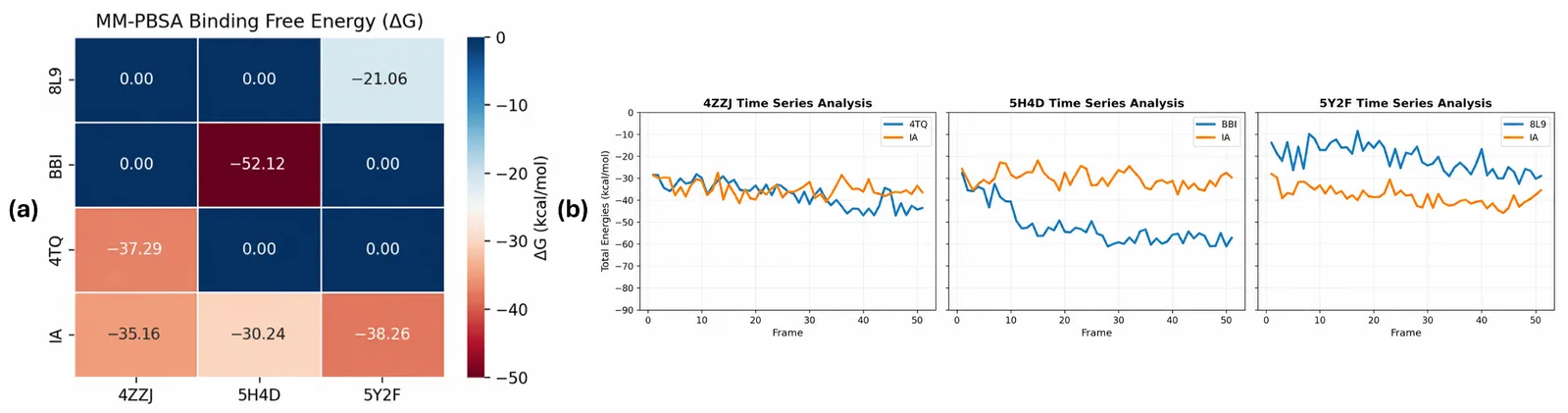
MM-PBSA-derived binding-energy analysis. (**a**) Heatmap of mean MM-PBSA binding-energy estimates and (**b**) frame-wise variation in total MM-PBSA energy across 51 analyzed snapshots.

Molecular mechanics Poisson–Boltzmann surface area (MM-PBSA) analysis was performed to evaluate the interaction energetics of incensole acetate (IA) toward SIRT1, SIRT3, and SIRT6 and to compare resulting energetic profiles with those of corresponding reference ligands 4TQ, BBI, and 8L9, respectively. Mean total MM-PBSA-derived binding-energy estimates calculated across 51 frames revealed distinct energetic profiles among the three sirtuin complexes (Figure 3).

For SIRT1 (4ZZJ), the reference ligand 4TQ exhibited a mean total MM-PBSA-derived binding-energy estimate of −37.29 kcal/mol, whereas IA showed a slightly less favorable value of −35.16 kcal/mol. Thus, IA displayed MM-PBSA energetics comparable to that of 4TQ, although the reference ligand maintained a modest energetic advantage. Analysis of the frame-by-frame energy profile further showed that 4TQ tended to reach more negative energies during the later part of the trajectory, whereas IA remained predominantly within a narrower energy range. Consistent with this observation, the energetic fluctuations were lower for IA than for 4TQ (SD: 3.32 vs. 5.49 kcal/mol, respectively), indicating a comparatively more uniform energetic profile for the IA–SIRT1 complex. Energy decomposition further clarified the energetic contributions underlying these differences. For SIRT1, IA exhibited a slightly more favorable van der Waals contribution than 4TQ (−46.63 ± 2.35 vs. −44.25 ± 7.94 kcal/mol), whereas its polar-solvation contribution was less favorable (16.92 ± 3.80 vs. 11.98 ± 4.43 kcal/mol) (Supplementary Table S1).

A more pronounced difference was observed for SIRT3 (5H4D). BBI exhibited the most favorable mean total MM-PBSA-derived binding-energy estimate among the reference complexes, with a mean total energy of −52.12 kcal/mol compared with −30.24 kcal/mol for IA. The time-series analysis showed that BBI progressively shifted toward substantially more negative energy values and remained predominantly around the −50 to −60 kcal/mol region during the later frames. However, BBI also exhibited greater energetic fluctuations than IA, with SD values of approximately 8.61 and 3.63 kcal/mol, respectively. Therefore, although BBI displayed considerably more favorable binding energetics toward SIRT3, IA showed a less fluctuating energy profile throughout the analyzed frames. For SIRT3, BBI showed a markedly more favorable van der Waals contribution than IA (−62.72 ± 9.16 vs. −41.06 ± 4.19 kcal/mol), which represented the principal energetic difference contributing to the more favorable total MM-PBSA energy of BBI (Supplementary Table S1).

In contrast, IA exhibited markedly more favorable MM-PBSA energetics toward SIRT6 (5Y2F) than the reference ligand 8L9. The mean total MM-PBSA-derived binding energy was −38.26 kcal/mol for IA compared with only −21.06 kcal/mol for 8L9. This difference was consistently reflected in the time-series analysis, in which the IA–SIRT6 complex maintained more negative total energy values throughout most of the trajectory. Furthermore, IA showed substantially lower relative energetic variability than 8L9 (SD values 3.90 vs 6.16 kcal/mol respectively), supporting a more consistent energetic profile for IA in the SIRT6 binding site. In the SIRT6 system, 8L9 exhibited a strong favorable electrostatic contribution (−53.18 ± 27.92 kcal/mol); however, this was largely counterbalanced by a pronounced unfavorable polar-solvation contribution (67.15 ± 25.38 kcal/mol). In contrast, IA displayed a substantially more favorable van der Waals contribution (−50.96 ± 3.83 kcal/mol) together with a markedly smaller polar-solvation penalty (17.61 ± 2.35 kcal/mol), resulting in the more favorable total binding-energy estimate for the SIRT6–IA complex (Supplementary Table S1).

Block-based statistical analysis of total MM-PBSA energies revealed no significant difference between SIRT1–4TQ and SIRT1–IA (*p* = 0.260). In contrast, significant differences were observed between SIRT3–BBI and SIRT3–IA and between SIRT6–8L9 and SIRT6–IA (*p* < 0.001 for both comparisons).

### 2.5. Protein–Protein Interactions

**Figure 4:**
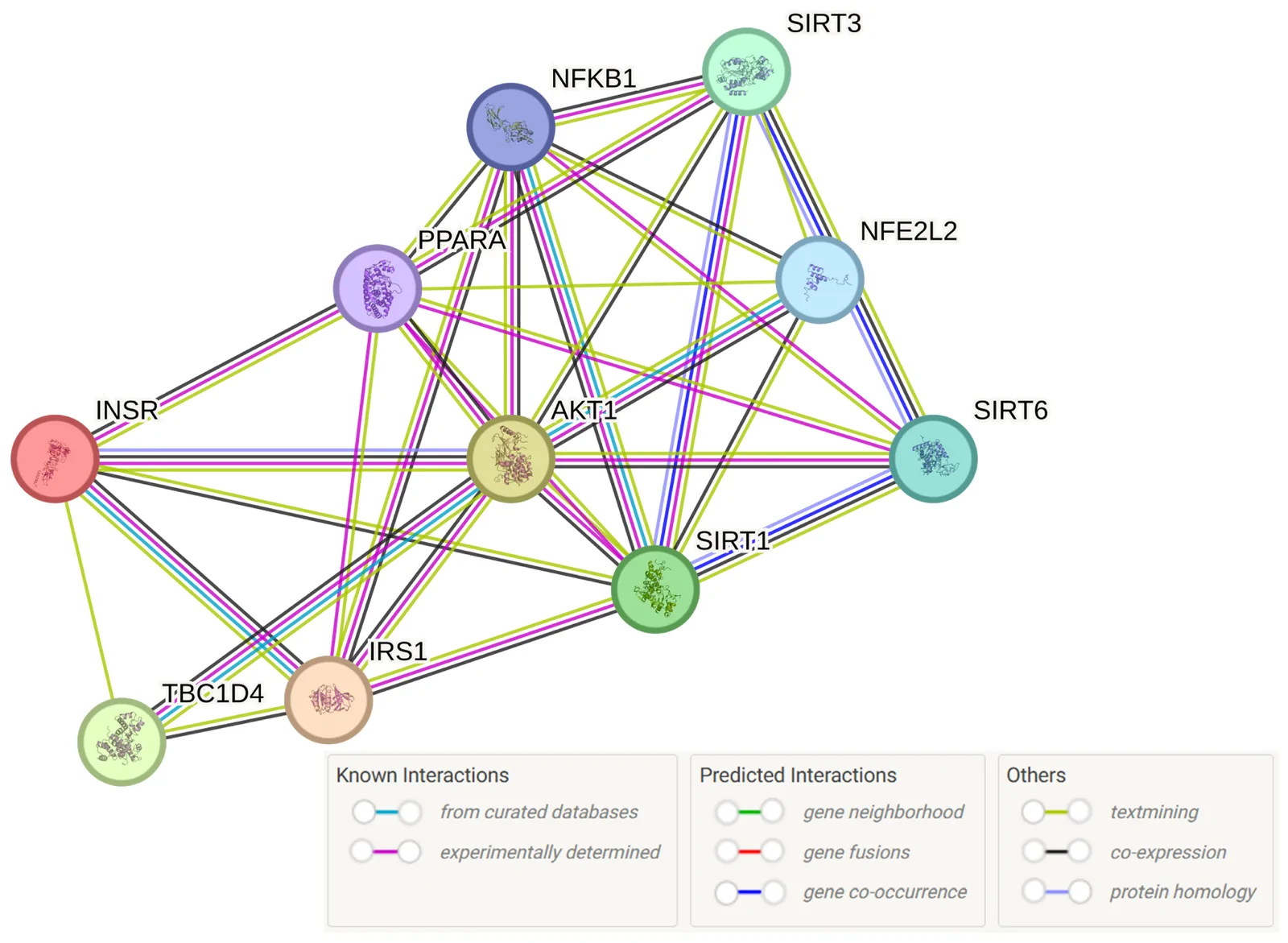
STRING protein–protein interaction analysis of insulin signaling pathway, sirtuins, NF-κB and NRF2 (INSR: insulin receptor, IRS1: insulin receptor substrate 1, AKT: protein kinase B, TBCD1D4/AS160: AKT substrate of 160 kDa, SIRT1: sirtuin 1, SIRT3: sirtuin 3, SIRT6: sirtuin 6, NFKB1/NFkB: nuclear factor kappa B, NFE2L2/NRF2: nuclear factor erythroid 2-related factor 2, PPARA/PPARa: peroxisome proliferator-activated receptor alpha).

Protein–protein interaction analysis of the insulin signaling pathway, sirtuins, NF-κB and NRF2 revealed that highly interconnected interactions were present. Several proteins exhibited a high number of interactions, AKT with eight interactions, forming central nodes might play key regulatory roles. The absence of isolated nodes following network filtering further supports the biological relevance and internal consistency of the selected protein group (Figure 4).

### 2.6. Characterization of Neuronal Differentiation

**Figure 5:**
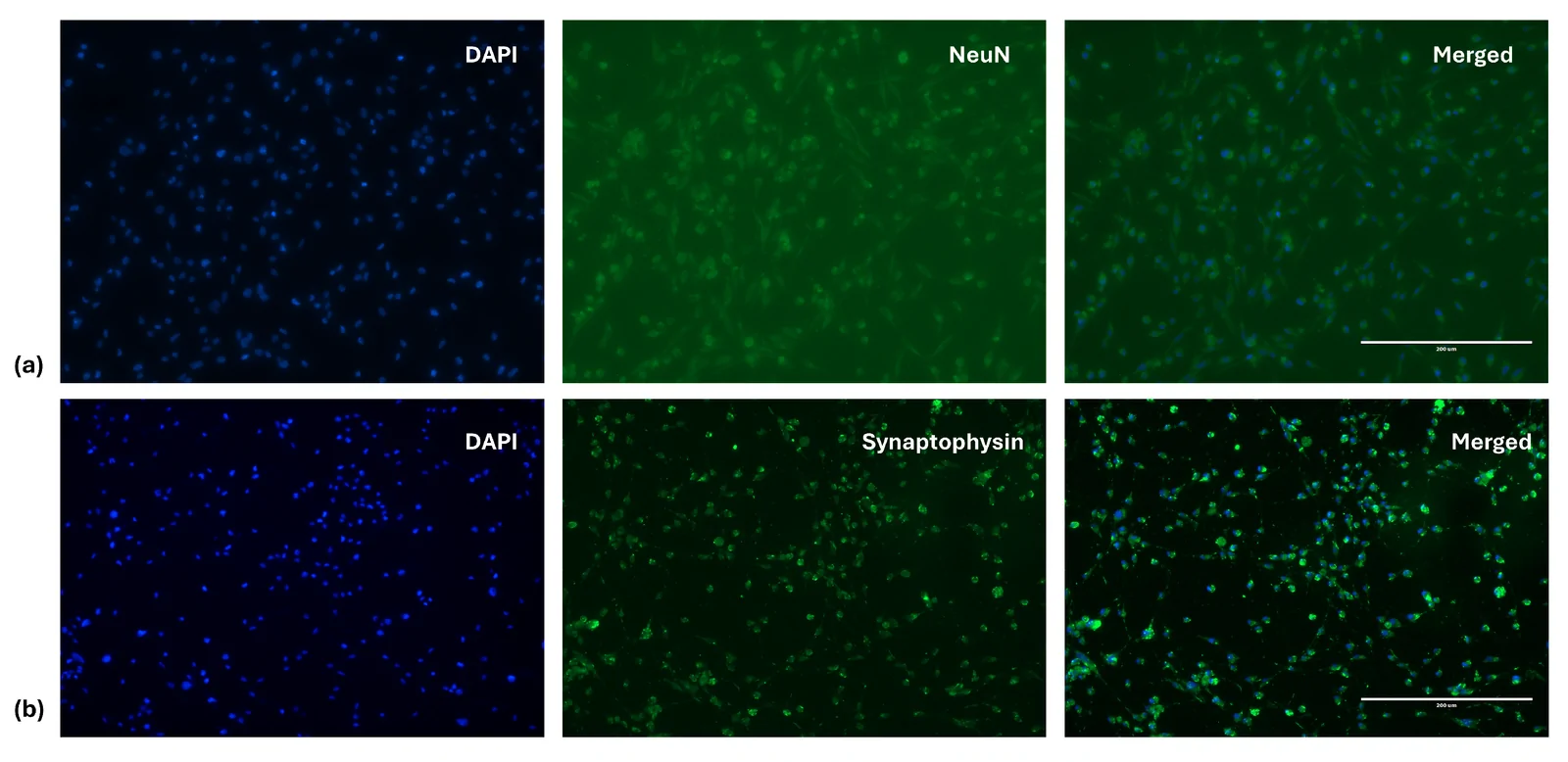
Neuronal differentiations characterization of human bone marrow-derived mesenchymal stem cell (**a**) NeuN (green) and (**b**) synaptophysin (green) with DAPI (blue) staining (EVOS FL, Invitrogen. The scale bars equal to 200 µm).

BM-MSCs differentiated toward a neuronal phenotype exhibited immunoreactivity for both NeuN and synaptophysin (Figure 5). Quantitative analysis showed that 75.3% of the analyzed cells were NeuN positive, and 69.5% were synaptophysin positive. These findings quantitatively support neuronal differentiation of the BM-MSC-derived cells.

### 2.7. MTT Assay Results

**Figure 6:**
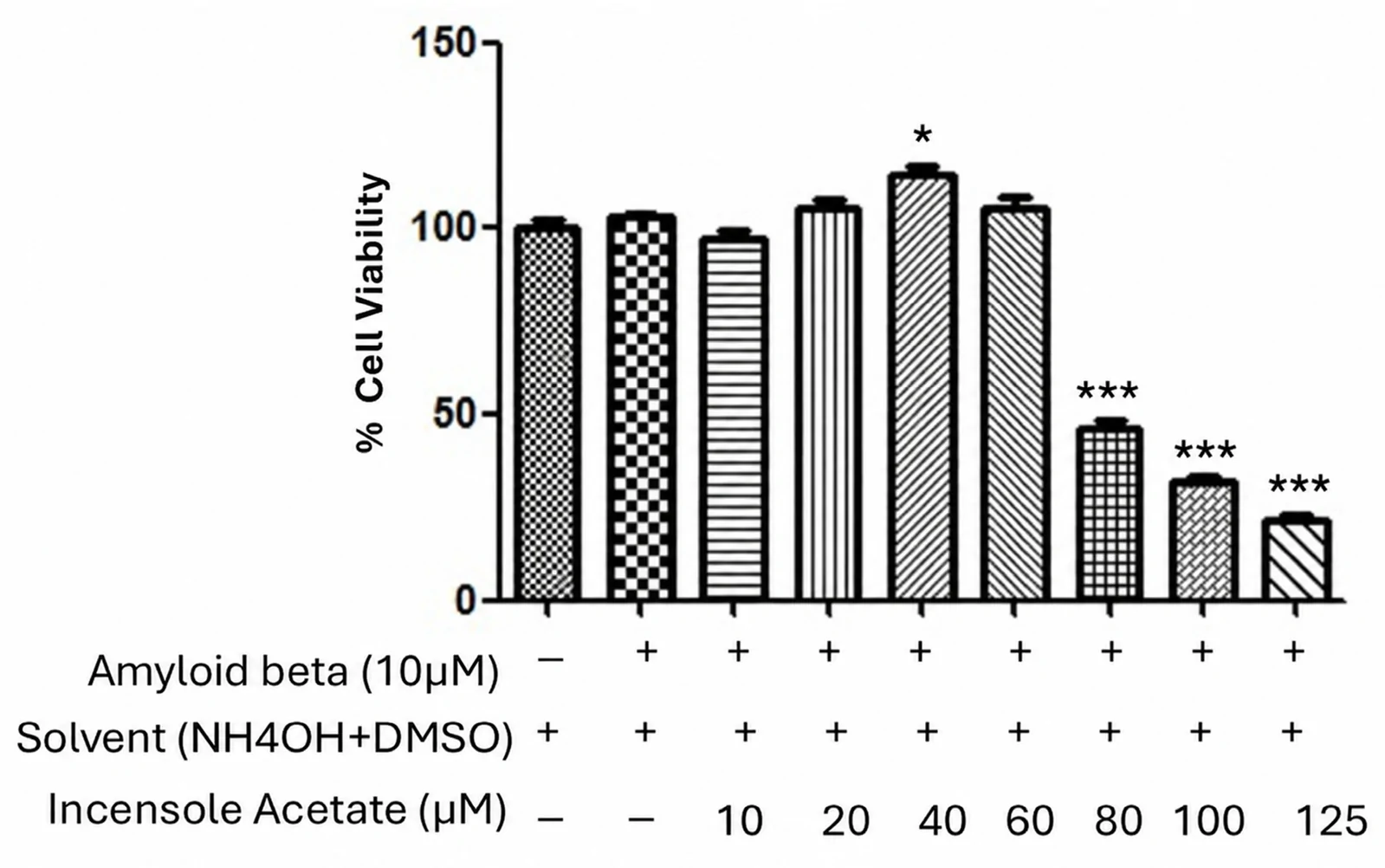
Effect of incensole acetate on cell viability in differentiated BM-MSC treated with amyloid beta 1–42. Statistical analyses were performed with one-way ANOVA and Tukey’s multiple comparison test; statistical significance is depicted as * *p* < 0.05, *** *p* < 0.001 compared to the vehicle.

A non-cytotoxic working concentration of IA was selected based on the MTT cell-viability assay. According to our results, a 40 µM dose of incensole acetate significantly increased cell viability within the experimental AD model, while 80 µM, 100 µM and 125 µM doses showed inhibitory effects on cellular survival compared to the vehicle control (Figure 6). Therefore, the following experiments were conducted with a 40 µM dose of IA (final DMSO concentration is 0.0278% *v*/*v*).

### 2.8. Gene Expressions of Sirtuin Family Proteins

SIRT1, SIRT3 and SIRT6 protein expression levels were determined besides in silico results.

**Figure 7:**
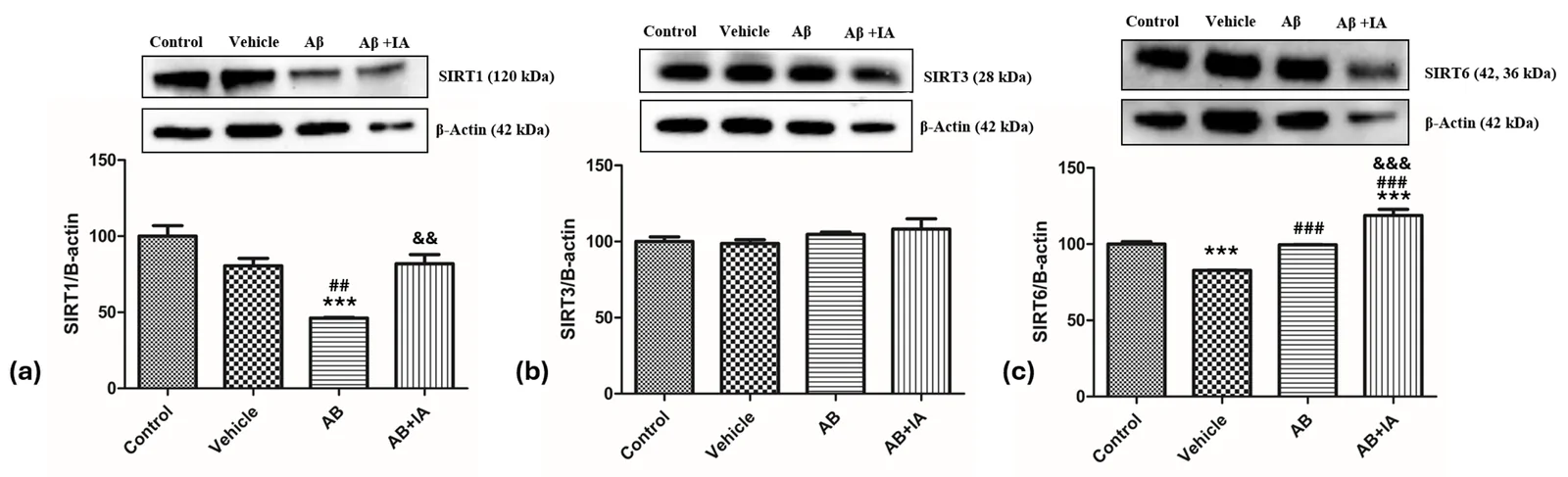
Western blot analysis of (**a**) SIRT1/B-actin, (**b**) SIRT3/B-Actin and (**c**) SIRT6/B-actin. Statistical analyses were performed with one-way ANOVA and Tukey’s multiple comparison test; statistical significance is depicted as *** *p* < 0.001 compared to the control, ## *p* < 0.01, ### *p* < 0.001 compared to the vehicle, && *p* < 0.01, &&& *p* < 0.001 compared to the Aβ1–42 group.

Aβ treatment decreased SIRT1 expression significantly, while IA treatment increased its expression significantly compared to the Aβ group and made it closer to the basal level (Figure 7a).

There was no significant change within SIRT3 protein level among groups. However, IA treatment increased its expression compared to the Aβ group nonsignificant (Figure 7b).

SIRT6 protein expression was significantly higher in the IA group compared to all groups (Figure 7c).

### 2.9. Gene Expressions of Insulin Signaling Pathway Proteins

**Figure 8:**
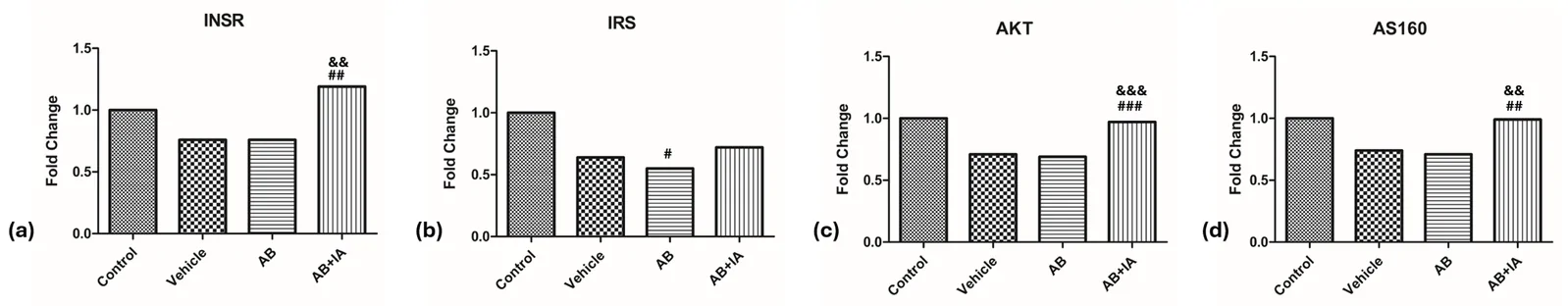
Insulin signaling pathway gene expression in mRNA levels determined by RT-qPCR analyses. (**a**) INSR mRNA expression; (**b**) IRS1 mRNA expression; (**c**) AKT mRNA expression; (**d**) AS160 mRNA expression. Statistical analyses were performed with one-way ANOVA and Tukey’s multiple comparison test; statistical significance is indicated as # *p* < 0.05, ## *p* < 0.01, ### *p* < 0.001 compared to the vehicle, && *p* < 0.01, &&& *p* < 0.001 compared to the Aβ1–42 group.

**Figure 9:**
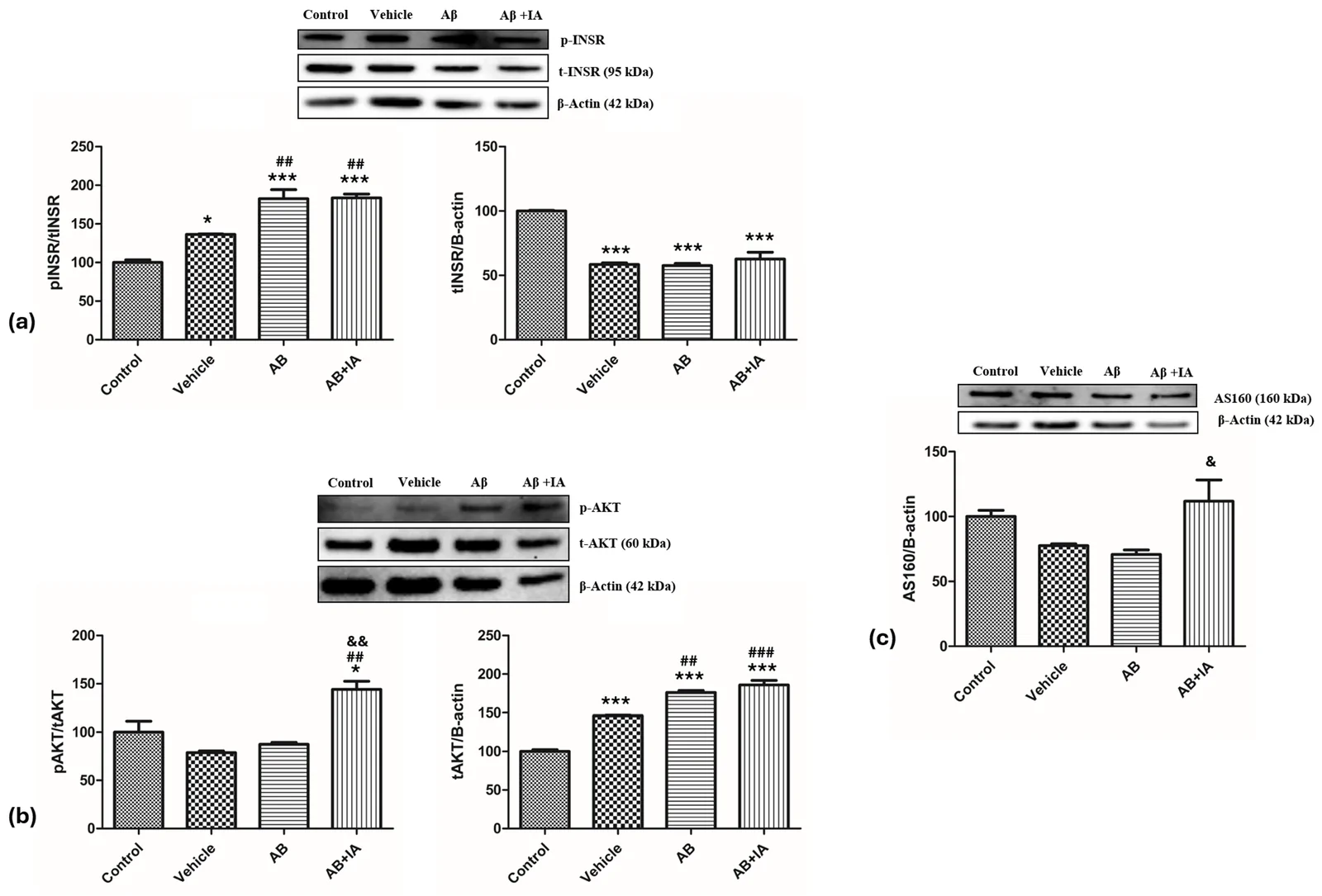
Western blot analysis of insulin signaling pathway proteins (**a**) p-INSR/t-INSR and t-INSR/B-actin, (**b**) p-AKT/t-AKT and t-AKT/B-actin and (**c**) AS160/B-actin. Statistical analyses were performed with one-way ANOVA and Tukey’s multiple comparison test; statistical significance is depicted as * *p* < 0.05, *** *p* < 0.001 compared to the control, ## *p* < 0.01, ### *p* < 0.001 compared to the vehicle, & *p* < 0.05 and && *p* < 0.01 compared to the Aβ1–42 group.

INSR, AKT, and AS160 were evaluated at both the mRNA and protein levels, whereas IRS was assessed only at the mRNA level. According to the findings, INSR mRNA level was significantly increased in the IA treatment group (Figure 8a), while p-INSR was increased in both Aβ and Aβ+IA groups (Figure 9a). However, t-INSR level, which normally decreases in Alzheimer’s disease [11], decreased in the Aβ group, and a slight increment was observed in IA treatment (Figure 9a).

It was determined that the IRS decreased at a statistically significant level in the Aβ experimental group compared to the control (Figure 8b). The expression level of AKT in mRNA level was significantly high in the IA group compared to vehicle and Aβ level (Figure 8c). Similarly, pAKT level was significantly increased with IA treatments (Figure 9b). t-AKT levels were increased in all three groups compared to the control, where a significant increase in Aβ and Aβ+IA groups compared to the vehicle was also observed (Figure 9b). When the expression of the AS160 gene was examined, it was seen that it increased significantly in the IA treatment group, both in mRNA compared to the vehicle and Aβ groups (Figure 8d) and protein levels compared to Aβ groups (Figure 9c).

### 2.10. Protein Expressions of Aβ, Nf-κB and NRF2 Proteins

**Figure 10:**
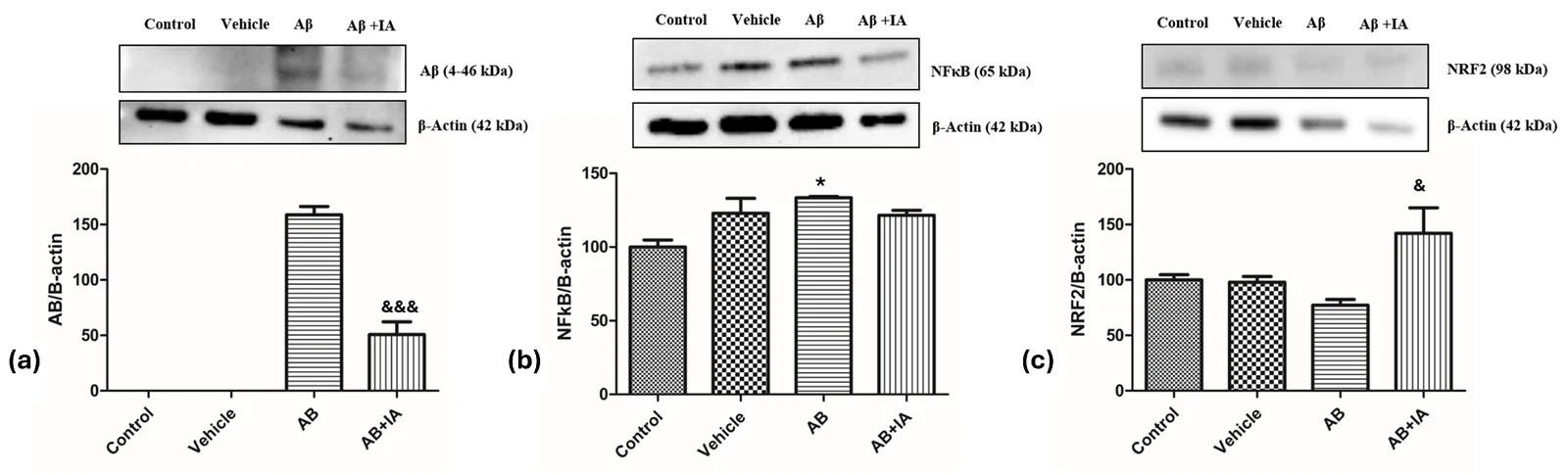
Western blot analysis of (**a**) AB/B-actin, (**b**) NF-kB/B-actin and (**c**) NRF2/B-actin. Statistical analyses were performed with one-way ANOVA and Tukey’s multiple comparison test; statistical significance is depicted as * *p* < 0.05 compared to the control, & *p* < 0.05 and &&& *p* < 0.001 compared to the Aβ1–42 group.

Western blot analysis detected Aβ immunoreactivity in the Aβ and Aβ+IA groups. The relative Aβ band intensity was significantly lower in the Aβ+IA group compared with the Aβ group (Figure 10a). NF-κB protein level, which is an indicator of inflammation, significantly increased in the amyloid beta group, where IA treatment decreased its expression close to the basal level (Figure 10b). It was determined that transcription factor NRF2 increased significantly by IA treatment, whereas Aβ toxicity caused a nonsignificant decrement (Figure 10c).

### 2.11. Immunofluorescent Staining of PPARα

**Figure 11:**
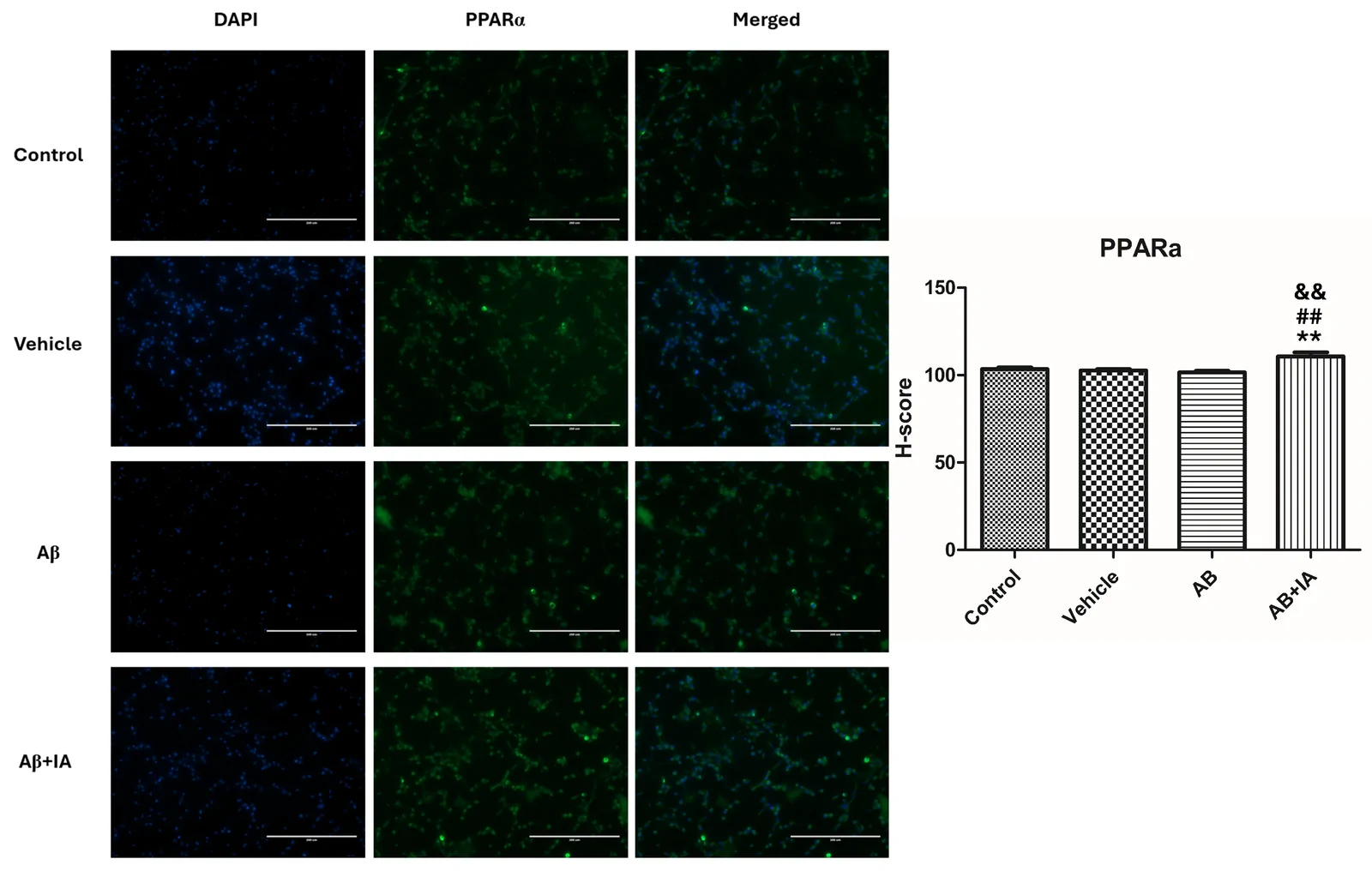
Immunofluorescent staining of PPARα. Representative images of PPARα (green) and DAPI (blue). Semi-quantitative H-score analyses were conducted by Image J Fiji. Statistical analyses were performed with one-way ANOVA and Tukey’s multiple comparison test; statistical significance is depicted as ** *p* < 0.01 compared to the control, ## *p* < 0.01 compared to the vehicle, && *p* < 0.01 compared to the Aβ1–42 group (EVOS FL, Invitrogen. The scale bars equal to 200 µm).

PPARα, a transcription factor that controls lipid metabolism, expression levels were determined by immunofluorescent staining. As shown in Figure 11, IA treatment significantly increased PPARα expression compared to all three groups.

## 3. Discussion

With increasing life expectancy, diseases related to senility have been increasing worldwide. One of the most significant of these diseases, AD, ranks among the leading causes of death. From a neuropathological perspective, senile amyloid beta plaques and hyperphosphorylated tau that form neurofibrillary tangles are two fundamental causes of AD. However, diabetes, cardiovascular diseases, hypertension, genetic factors, and low educational level contribute to the process of disease formation and the multifactorial aspects of the disease. The fact that AD currently has no preventive or disease-modifying treatment and that the existing treatments are aimed at alleviating symptoms makes AD a significant topic of research today [2,3,28].

Insulin resistance appears due to the cells’ inadequate response to insulin causing hyperinsulinemia and hyperglycemia, as seen in diabetes mellitus, obesity, metabolic and neurodegenerative disorders [29]. Increased insulin levels reduce insulin degrading enzyme (IDE) activity, which impairs amyloid beta degradation and increases amyloid beta aggregation. Meanwhile, amyloid beta binds to IR and IGFR, shows an antagonistic effect, and creates an inflammatory response [30].

In this study, ADMET analyses were first performed to characterize the predicted pharmacokinetic, metabolic, and toxicity profiles of incensole acetate (IA). Subsequently, the potential interactions of IA and the corresponding reference ligands with SIRT1, SIRT3, and SIRT6 were investigated using molecular docking, molecular dynamics simulations, and MM-PBSA analyses. For the in vitro part of this study, mesenchymal stem cells were differentiated toward a neuronal phenotype, followed by establishment of an Aβ1–42-induced cellular AD model. A non-cytotoxic working concentration of IA was then selected based on the MTT cell-viability assay. RNA and protein were isolated from the experimental groups for subsequent molecular analyses. INSR, AKT, and AS160 were evaluated at both the mRNA and protein levels, whereas IRS was quantitatively evaluated only at the mRNA level because a reliable IRS signal suitable for Western blot quantification could not be obtained. SIRT1, SIRT3, SIRT6, NF-κB, and NRF2 protein levels were assessed by Western blotting, whereas PPARα expression was evaluated by immunofluorescence staining.

As a result of ADMET analysis of IA, it has been predicted in silico that the gastrointestinal absorption and blood–brain barrier (BBB) permeability are high. For a candidate drug molecule to be used in AD treatment, it is known that it should be BBB permeant [31]. Despite its favorable predicted gastrointestinal absorption and blood–brain barrier permeability, the in silico safety profile of IA also revealed potential liabilities. IA showed predicted H-HT and DILI probabilities of 0.729 and 0.766, respectively, indicating that possible hepatic toxicity should be considered during further development. Although these values were lower than those predicted for the positive toxic/carcinogenic control AFB1, they cannot be interpreted as evidence of hepatic safety. In addition, IA was predicted to inhibit CYP2C9 and CYP3A4. Inhibition of these metabolic enzymes may alter the clearance of co-administered compounds that depend on these CYP pathways, potentially increasing systemic exposure and the risk of drug–drug interactions. Importantly, these predictions are computational and do not establish that IA causes hepatotoxicity or clinically relevant CYP inhibition. Therefore, dedicated experimental studies, including CYP inhibition assays, hepatocyte-based toxicity testing, and in vivo pharmacokinetic and hepatic safety evaluations, will be necessary before the translational potential of IA can be established. These pharmacokinetic characteristics support further investigation of IA as a candidate for CNS-targeted applications; however, its predicted hepatic and CYP-related safety liabilities should also be considered.

As a fundamental method of in silico drug design studies, molecular docking predicts the molecular interaction of a molecule and a protein at the atomic level. Therefore, the binding capacity of drug candidate molecules to the target proteins can be determined, and drug design studies could be accelerated [32]. Molecular docking analysis demonstrated that IA was able to interact with the investigated binding regions of all three sirtuins, although its predicted binding affinities were lower than those of the corresponding reference ligands. Among the three targets, IA showed the most favorable docking score toward SIRT3, where its affinity was relatively close to that of BBI. IA also shared several binding-site interactions with reference ligands. In SIRT1, IA and 4TQ both interacted with ILE41 and ILE45; in SIRT3, both IA and BBI formed a hydrogen bond with ARG158 and shared hydrophobic interactions with PHE157, PHE180, ILE230, and HIS248; and in SIRT6, IA and 8L9 shared hydrophobic contacts with VAL68, PRO78, and PHE80, while IA additionally formed a hydrogen bond with PHE84. These overlapping residues suggest that IA can occupy interaction environments similar to those of the reference ligands, particularly in SIRT3. However, the subsequent MD and MM-PBSA analyses demonstrated that the energetic behavior of these complexes under dynamic conditions did not necessarily follow the initial docking-score ranking, emphasizing the complementary nature of static docking and trajectory-based binding-energy analyses [33,34].

MD simulations further provided comparative information on the dynamic behavior of IA and the corresponding reference ligands in complexes with the three sirtuins. For SIRT1, IA exhibited lower mean RMSD and RMSF values than 4TQ and maintained a relatively compact Rg profile. However, neither SIRT1 complex displayed a single persistent equilibrium plateau throughout the complete 100 ns trajectory, with both systems showing time-dependent conformational changes toward the later stages of the simulation. Despite these differences, the MM-PBSA-derived binding-energy estimate of IA (−35.16 ± 3.32 kcal/mol) approached that of 4TQ (−37.29 ± 5.49 kcal/mol), suggesting energetically favorable IA–SIRT1 interactions under the simulated conditions. Ligand RMSD analysis showed a lower positional deviation for 4TQ than for IA (0.13 ± 0.05 vs. 0.22 ± 0.05 nm).

For SIRT3, IA exhibited lower mean protein RMSD and RMSF values than BBI, while both complexes maintained relatively narrow Rg profiles, indicating preservation of overall protein compactness during the simulation. The two complexes nevertheless showed different temporal behaviors: SIRT3–BBI reached a relatively stable RMSD plateau after approximately 20 ns, whereas SIRT3–IA underwent a more gradual conformational adjustment and displayed comparatively stable behavior during the later stages of the trajectory. Despite these structural characteristics, BBI showed substantially more favorable MM-PBSA-derived binding energetics than IA (−52.12 ± 8.61 vs. −30.24 ± 3.63 kcal/mol), although IA exhibited lower energetic variability. Ligand RMSD analysis also showed a lower positional deviation for IA than for BBI (0.12 ± 0.04 vs. 0.17 ± 0.07 nm). The average number of protein–ligand hydrogen bonds was low for both complexes, although slightly higher for IA than for BBI (0.09 ± 0.29 vs. 0.002 ± 0.04), suggesting that persistent hydrogen bonding was not a major feature of either SIRT3 complex. Thus, the lower structural and positional deviations observed for IA did not translate into more favorable MM-PBSA energetics relative to BBI.

The most notable relative energetic difference was observed for SIRT6, where IA showed a substantially more favorable MM-PBSA-derived binding-energy estimate (−38.26 ± 3.90 kcal/mol) than 8L9 (−21.06 ± 6.16 kcal/mol) and maintained more negative energies throughout most of the analyzed trajectory. IA also exhibited a lower ligand RMSD than 8L9 (0.10 ± 0.02 vs. 0.25 ± 0.05 nm), indicating a lower positional deviation during the simulation. In contrast, SIRT6–8L9 showed a markedly higher average number of protein–ligand hydrogen bonds than SIRT6–IA. This difference indicates that hydrogen-bond frequency alone does not account for the overall interaction energetics, which also reflect van der Waals, electrostatic, polar-solvation, and non-polar-solvation contributions. The comparable Rg profiles of the two SIRT6 complexes further indicated preservation of overall protein compactness without major global expansion or collapse. Taken together, these findings provide comparative structural and energetic evidence for IA–sirtuin interactions, with SIRT6 showing the most favorable relative MM-PBSA energetic profile for IA compared with its corresponding reference ligand under the simulated conditions.

Although the 100 ns simulations provided comparative information on the structural behavior of IA and the corresponding reference complexes, each system was represented by a single production trajectory and therefore does not provide exhaustive conformational sampling. Longer simulations and independent replicate trajectories initiated from different random seeds would provide broader conformational sampling and greater statistical confidence. Accordingly, the present MD findings should be interpreted as comparative structural analyses rather than definitive evidence of long-term conformational convergence.

The protein–protein interaction (PPI) network further supported the functional interconnection between sirtuins and the signaling pathways investigated in this study. SIRT1, SIRT3, and SIRT6 were integrated into a highly interconnected network involving key regulators of insulin signaling (INSR, IRS1, AKT1, and AS160), oxidative stress (NRF2), inflammation (NF-κB), and metabolic regulation (PPARα). Notably, the extensive connectivity among these proteins suggests that sirtuin-associated effects may extend beyond individual targets and converge on coordinated regulation of metabolic, inflammatory, and oxidative-stress responses. However, these network associations should be interpreted as evidence of functional connectivity rather than direct causal or physical interactions between all protein pairs.

As part of the in vitro analyses, firstly, mesenchymal stem cells were differentiated into neurons. MSCs are multipotent stem cells and can be found in several tissues like bone marrow, placenta, endometrium, and adipose tissue. They can be differentiated into osteoblasts, chondroblasts, adipocytes, and neurons. They offer several advantages over other stem cell types, including ease of culturing, a low immune response, and the ability to differentiate easily. In recent years, mesenchymal stem cells have been used for modeling and treatment of neurodegenerative disorders [35,36].

Generally, as an in vitro AD model, neuroblastoma cells are used [37]. However, because they are cancer cells, they cannot fully reflect neuronal properties. Therefore, in this study, MSC-derived neuron cells were used as a model, and differentiation was shown by the neuronal markers NeuN and synaptophysin. For establishing an experimental AD model, a 10 µM Aβ_1–42_ peptide dose, which was employed in our previous studies, was used [38,39]. To determine a non-toxic dose of incensole acetate, MTT tests with different doses were conducted, and a 40 µM dose was chosen due to increased cell viability. In previous studies in the C6 glioma cell line [27] and in olfactory bulbus neural stem cells [26], a 100 µM IA dose was used; this difference was thought to stem from variations in cell lines. The 40 μM IA concentration used in the present study was selected based on cell-viability findings and should not be interpreted as an optimized molecularly active dose.

Insulin signaling pathway components were examined at both the transcript and protein levels. INSR mRNA expression was significantly increased in the Aβ+IA group compared with the vehicle and Aβ groups. At the protein level, p-INSR levels were increased in both the Aβ and Aβ+IA groups compared with the control, with a greater increase in the Aβ+IA group. In contrast, total INSR levels were reduced following Aβ treatment, with a more pronounced decrease in the Aβ group than in the Aβ+IA group. In previous studies, INSR levels in AD patients were found to be significantly decreased [11,40,41,42], which is parallel to our results. Importantly, the increase in INSR mRNA following IA treatment was not fully paralleled by changes in total INSR protein. Such discrepancies between transcript and protein abundance are not unexpected, as protein levels are additionally influenced by mRNA stability, translational regulation, protein turnover, and post-translational mechanisms [43]. Therefore, the increased INSR transcript level following IA treatment should be interpreted as evidence of modulation at the transcriptional level rather than as a direct indication of increased receptor abundance or activity.

Our results showed that IRS mRNA levels were significantly reduced in the Aβ group, while a nonsignificant increase in the Aβ+IA group compared to the Aβ group was observed. IRS levels decrease when insulin resistance occurs [11,14,44,45], which supports our findings. Although the increase following IA treatment did not reach statistical significance, this trend may indicate a partial modulation of IRS expression under the experimental conditions. Further studies are required to determine whether this effect translates into functional improvement of insulin signaling.

A similar distinction between transcript abundance, total protein level, and phosphorylation status was observed for AKT. IA treatment significantly increased AKT mRNA expression compared with the vehicle and Aβ groups, whereas total AKT protein was increased in both the Aβ and Aβ+IA groups. In contrast, p-AKT was significantly increased following IA treatment compared with both the vehicle and Aβ groups. These findings demonstrate that changes in AKT transcript abundance do not necessarily parallel total AKT protein levels and emphasize that phosphorylation represents an additional regulatory layer reflecting signaling state rather than protein abundance. In the former studies, pAKT levels were found to decrease in AD patients and in the case of insulin resistance [44,46,47]. Therefore, the increase in p-AKT following IA treatment is consistent with enhanced AKT signaling and suggests that IA may favorably modulate downstream components of the insulin signaling pathway. However, this increase cannot be attributed exclusively to INSR activation, since AKT integrates signals from multiple upstream signaling pathways [48].

AS160 is a downstream component of the AKT signaling pathway and plays an important role in the regulation of GLUT4 trafficking and glucose metabolism [49]. In the present study, IA treatment increased AS160 expression at both the mRNA and protein levels, accompanied by an increase in pAKT. Taken together, these findings suggest that IA may favorably modulate downstream components of the insulin signaling pathway. However, the observed increase in total AS160 should be interpreted as supportive of pathway modulation rather than as direct evidence of enhanced GLUT4-mediated glucose uptake.

SIRT1 induces insulin secretion from the pancreas, increases glucose uptake, and activates IRS1 and AKT while decreasing NF-κB activity [50]. When our results were examined, it was observed that Aβ toxicity decreased SIRT1 expression both at mRNA and protein levels, whereas its expression increased significantly with IA treatment. Taken together with the computational findings, the increase in SIRT1 expression observed following IA treatment suggests a potential association between the computationally predicted IA–SIRT1 interaction and modulation of insulin signaling. However, because the present computational and expression-based analyses do not establish functional activation of SIRT1 or a causal role of SIRT1 in the observed insulin-signaling changes, this relationship should be interpreted as supportive rather than causal.

The decrease in the expression of SIRT3 causes insulin signaling impairment, insulin resistance and a decrease in IRS1 and AKT activation [17]. According to the data we obtained, SIRT3 levels were not statistically different. However, IA treatment caused a slight increase in the protein level compared to the Aβ group. Although SIRT3 expression was not significantly altered by IA treatment, the docking and MD analyses indicated that IA could maintain interactions within the investigated SIRT3 binding region. However, its docking and MM-PBSA energetics were less favorable than those of BBI. Therefore, the present findings support a potential molecular interaction with SIRT3 rather than direct functional activation.

Nucleus-localized SIRT6 plays a crucial role in regulating glucose homeostasis and insulin sensitivity while also suppressing NF-κB activity [17,18,51]. As indicated by our experimental results, IA increased SIRT6 expression compared to all three groups. Although its initial docking score was weaker than that of 8L9, subsequent MM-PBSA analysis showed more favorable binding energetics for IA under dynamic conditions. These complementary findings suggest a potential interaction of IA with SIRT6 and an association between IA treatment and increased SIRT6 expression; however, they do not establish direct functional activation of SIRT6 or demonstrate that SIRT6 mediates the downstream effects of IA.

NF-κB is a transcription factor that regulates the expressions of inflammation-related genes. In the case of insulin resistance and in AD patients’ cerebral cortices, its expression increases [14,52]. Parallel to these findings, our results showed that, in the Aβ group, NF-κB expression increased significantly, which suggests that our AD model might trigger inflammation. Also, the IA treatment brought NF-kB expression down to the level of the control group, which was parallel to the results of the study of Moussaief et al. [27]. These findings suggest that the reduction in NF-κB following IA treatment occurred in parallel with changes in SIRT1 and SIRT6 expression; however, a causal relationship between these observations cannot be established from the present experiments.

NRF2 induces cells to produce an oxidative-stress response by regulating antioxidant proteins’ expressions. Also, it decreases insulin resistance by activating IRS1 [14]. In the AD patients’ hippocampus, the level of NRF2 is reduced [53,54]. Our findings revealed a nonsignificant decrease in NRF2 expression in the Aβ group, while NRF2 levels significantly increased in the IA-treated group. These findings suggest that IA may modulate NRF2-associated stress-response pathways under Aβ-induced conditions.

Western blot analysis detected Aβ immunoreactivity in the Aβ and Aβ+IA groups, with significantly lower relative Aβ band intensity following IA treatment. This finding indicates a reduction in detectable Aβ levels under the present experimental conditions. However, Western blot analysis alone cannot determine whether this reduction results from decreased Aβ production, enhanced degradation or clearance, altered aggregation state, or changes in cellular uptake. Therefore, the present data should not be interpreted as evidence for a specific mechanism of Aβ reduction. Further studies evaluating Aβ-processing and clearance pathways, including insulin-degrading enzyme (IDE), will be required to clarify the underlying mechanism.

PPARα is a fatty acid and cholesterol metabolism regulating transcription factor; its expression is reduced in the STZ model of rat and in postmortem tissues of AD patients. PPARα, as an antioxidant and anti-inflammatory molecule, shows neuroprotective effects in AD [55,56,57]. As revealed by our experimental findings, IA increased its expression compared to all three groups. This change may contribute to the metabolic and stress-response effects associated with IA treatment; however, functional activation of PPARα was not directly assessed.

## 4. Materials and Methods

**Figure 12:**
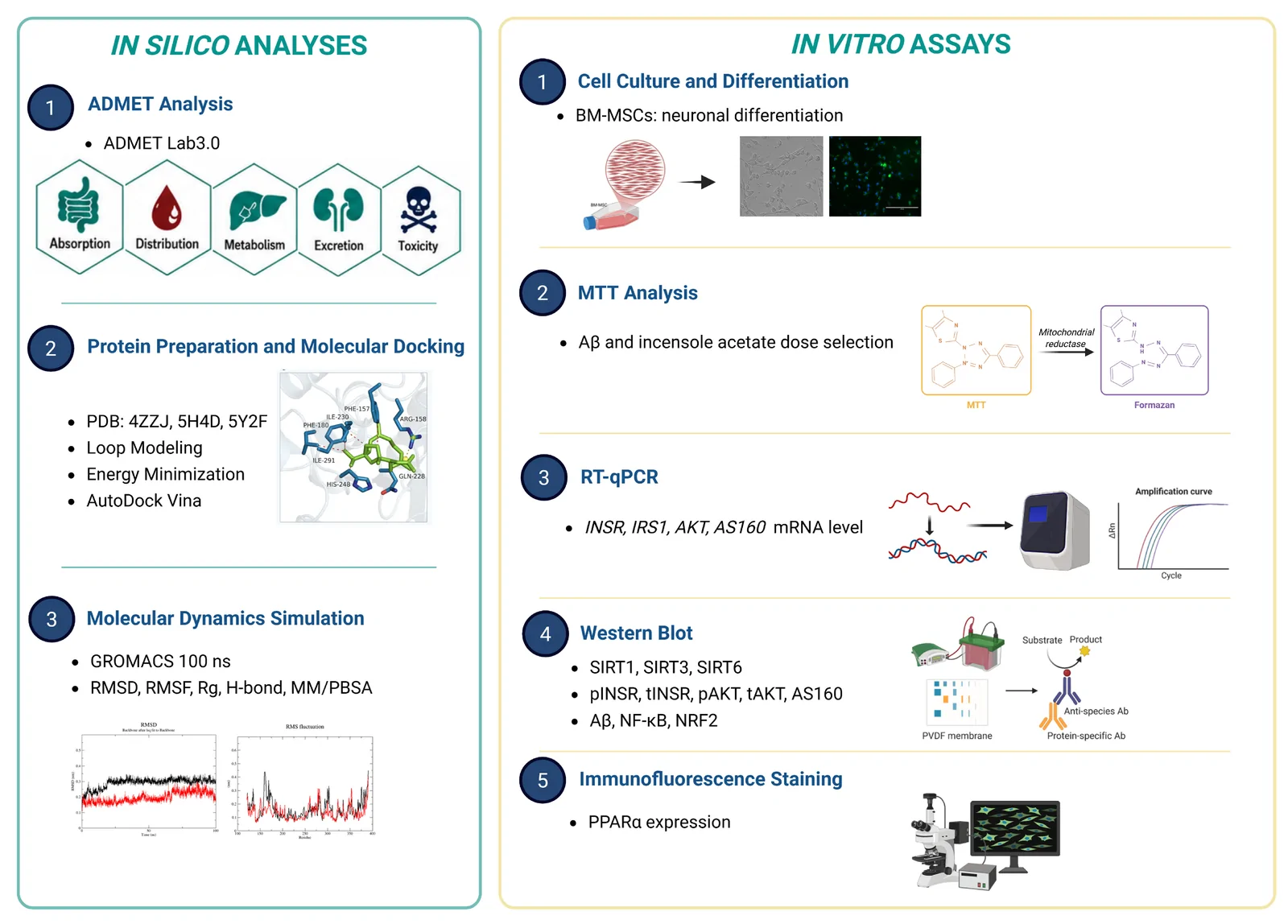
Schematic overview of computational analysis and in vitro studies employed in this study (Created in BioRender. Celik, U. (2026) https://BioRender.com/xx1hse4, accessed on 17 August 2026).

The in silico and in vitro workflows of this study are visualized in Figure 12.

### 4.1. ADMET Analyses

To predict the pharmacokinetic, metabolic and toxicity profiles of incensole acetate in silico, ADMET (Absorption, Distribution, Metabolism, Excretion, Toxicity) analysis was performed by web platforms SwissADME (https://www.swissadme.ch/, accessed on 25 November 2025) [58] and ADMETlab3.0 (https://admetlab3.scbdd.com/, accessed on 25 November 2025) [59]. Aflatoxin B1 (AFB1), a well-established toxic and carcinogenic compound, was included as a positive control for the toxicity prediction workflow.

### 4.2. Molecular Docking

Tertiary X-ray diffraction structures of proteins with their activators SIRT1/activator complex (PDB: 4ZZJ), SIRT3/amiodarone-HCl complex (PDB: 5H4D), and SIRT6/MDL-801 complex (PDB: 5Y2F) were obtained by utilizing the RCSB Protein Data Bank (https://www.rcsb.org/, accessed on 25 July 2025). The SMILE formats of the activators and incensole acetate (IA) were obtained from PubChem (https://pubchem.ncbi.nlm.nih.gov/, accessed on 25 July 2025), and their IDs were (4TQ (ID:381321908), BBI (ID:2157) 8L9 (ID: 134717373) and incensole acetate (ID: 53386731) respectively.

Molecular docking analyses were performed using AutoDock Vina (version 1.2.5) implemented in the SwissDock web server. For Vina calculations, ligand structures submitted as SMILES were automatically prepared through the SwissDock workflow and converted to PDBQT format using the mk_prepare_ligand.py script of Meeko (version 0.5.0). The receptor structures were converted to PDBQT format using the prepare_receptor tool of the AutoDockFR (ADFR) Suite (version 1.0). Ligand atom typing, partial-charge assignment, and rotatable-bond definitions were handled automatically during the ligand preparation procedure without manual modification. The standard Meeko preparation workflow uses Gasteiger partial charges.

A focused docking strategy was employed by defining a 20 Å × 20 Å × 20 Å search box around the predefined ligand binding region of each target. For SIRT1 (PDB: 4ZZJ), IA and the reference ligand 4TQ were docked using the loop-modeled prepared receptor, with the search box centered at x = 1.0, y = 46.0, and z = −1.0 Å, with a sampling exhaustiveness of 15. For SIRT3 (PDB: 5H4D), IA and the reference ligand BBI were docked using a search box centered at x = 18.0, y = 27.0, and z = −13.0 Å, with a sampling exhaustiveness of 30. For SIRT6 (PDB: 5Y2F), IA and the reference ligand 8L9 were docked using a search box centered at x = −2.0, y = 126.0, and z = 157.0 Å, with a sampling exhaustiveness of 13. For each target, IA and its corresponding reference ligand were evaluated using identical search-space and sampling settings to ensure direct comparability.

As an additional positive-control assessment of the docking protocol, the known SIRT1 inhibitor EX-527 (selisistat) was docked into the experimentally characterized inhibitor binding pocket of SIRT1 (PDB: 4I5I). Focused docking was performed using AutoDock Vina implemented in SwissDock with a 20 Å × 20 Å × 20 Å search box centered at x = 42, y = −21, and z = 18 Å, with a sampling exhaustiveness of 20. This analysis was performed solely to assess the ability of the docking workflow to identify a known SIRT1 ligand binding region and was not used for direct comparison with IA or the reference activator 4TQ.

### 4.3. Loop Modeling

**Figure 13:**
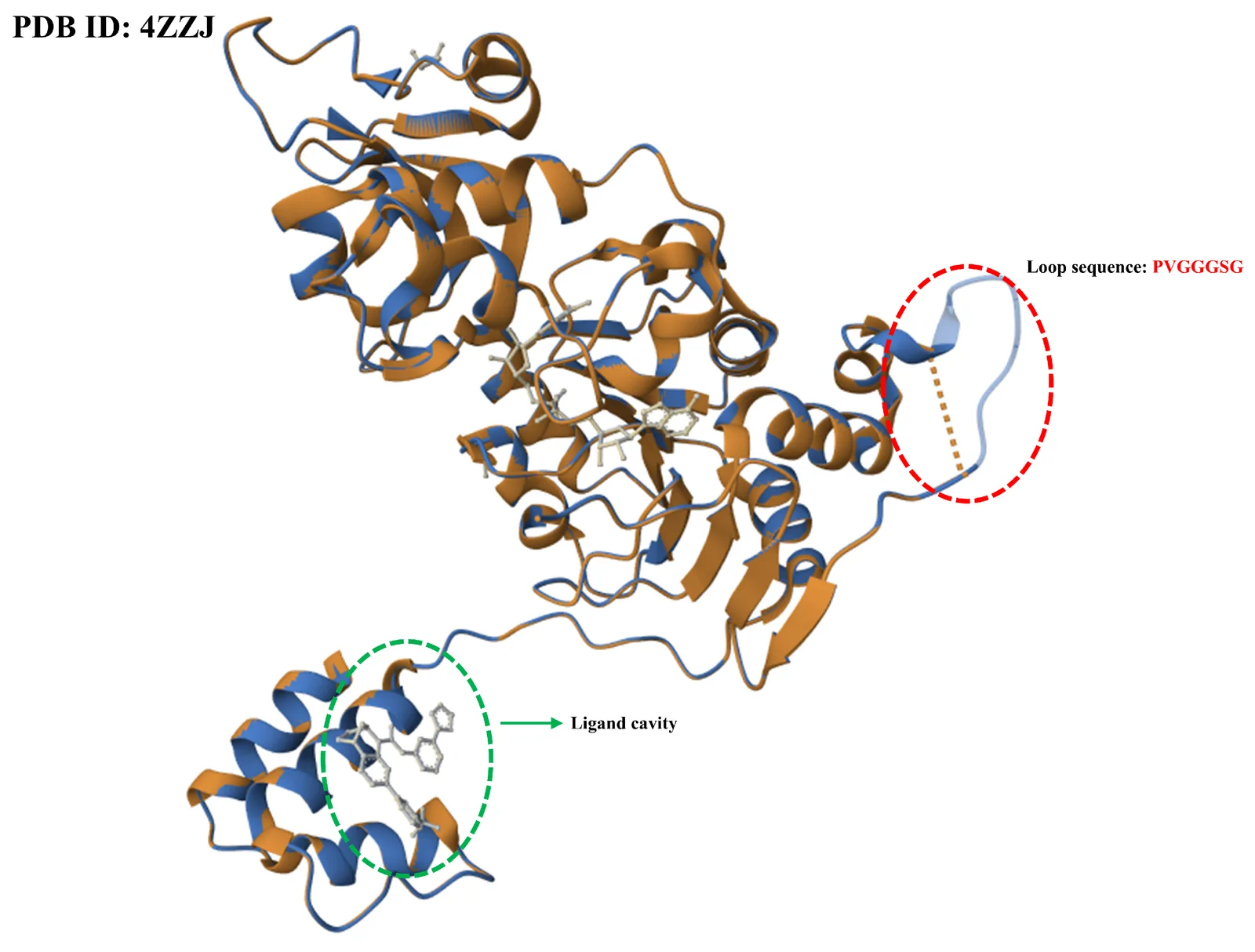
Loop modeling of gapped (brown) and looped (blue) protein via MODELLER. After structure alignment, TM scores and RMSD were 1 and 0.04 for 4ZZJ. Gaps and ligand cavities are indicated by red and green dashed circles respectively.

SIRT1 (PDB: 4ZZJ) protein was subjected to loop modeling by using MODELLER on Chimera 1.19 due to missing residues as our previous studies [60,61]. Missing residues were modeled based on the protein’s own structure, and the model with the lowest zDOPE (−1.29) and highest TM-score was selected for downstream analyses. Structural validation and alignment (Figure 13) were conducted within the RCSB Pairwise Structure Alignment tool (https://www.rcsb.org/alignment, accessed on 1 August 2025). In contrast, SIRT3 (PDB: 5H4D) contained no unresolved residues requiring reconstruction. Although SIRT6 (PDB: 5Y2F) contained unresolved residues at the terminal region, no internal gap was present within the structural region used for docking and subsequent MD simulations. Therefore, loop modeling was not considered necessary for SIRT3 or SIRT6.

### 4.4. Molecular Dynamic Simulations and MM-PBSA Analysis

For SIRT1, SIRT3 and SIRT6, the corresponding reference activator molecules and incensole acetate were subjected to 100 ns molecular dynamics simulations via SiBioLead LLP server (https://sibiolead.com/, accessed on 4 January 2026) utilizing GROMACS version 2021 [62] as our previous studies [60,61]. The simulations employed the OPLS-AA force field and the Simple Point Charge (SPC) water model. Ligand topologies and parameters were generated through the SiBioLead in-house workflow incorporating AMBERTOOLS and ACPYPE. All systems were solvated in a triclinic simulation box and neutralized with 0.15 M NaCl.

Energy minimization was performed using the steepest descent algorithm for a maximum of 5000 steps. The minimized systems were subsequently equilibrated for 100 ps under constant-volume and constant-temperature (NVT) conditions, followed by 100 ps under constant-pressure and constant-temperature (NPT) conditions. Temperature was maintained at 300 K using the V-rescale thermostat (τT = 0.1 ps). During NPT equilibration, isotropic pressure coupling at 1 bar was applied using the Berendsen barostat (τP = 2 ps). During the subsequent production simulations, pressure was controlled at 1 bar using the Parrinello–Rahman barostat with isotropic coupling (τP = 2 ps). Production simulations were performed for 100 ns using the leap-frog integrator with a 2 fs integration timestep.

Structural stability and dynamics were evaluated using protein backbone root mean square deviation (RMSD), root mean square fluctuation (RMSF), radius of gyration (Rg), ligand RMSD, and protein–ligand hydrogen-bond analyses. Trajectory stabilization was assessed from the time-dependent backbone RMSD profiles. A relatively stable or quasi-stable regime was operationally defined as a trajectory interval following the initial structural adjustment in which RMSD fluctuated around a relatively stable mean without a sustained directional increase or decrease. Because the complexes displayed different temporal behaviors, stabilization was evaluated individually for each protein–ligand system rather than by applying a single equilibration time to all trajectories.

MM-PBSA calculations were performed through the SiBioLead platform using the gmx_MM-PBSA approach [63] to estimate ligand–protein binding energetics. A total of 51 snapshots were included in the exported MM-PBSA output for each protein–ligand complex. The output files provided the total MM-PBSA energy together with its major energetic components, including van der Waals (VDWAALS), electrostatic (EEL), polar-solvation (EPB), and non-polar-solvation (ENPOLAR) terms. For each complex, the energetic components are reported as mean ± standard deviation (SD). The exported output files contained frame numbers but did not provide the corresponding simulation timestamps; therefore, the exact temporal interval between the 51 analyzed snapshots could not be independently verified. Entropic contributions were not reported in the exported output files and were therefore not included in the present analysis. Accordingly, the calculated values were interpreted as MM-PBSA-derived binding-energy estimates rather than absolute binding free energies.

For an additional statistical comparison of the total MM-PBSA energies, non-overlapping five-frame block averages were calculated to reduce the influence of temporal correlation between adjacent snapshots. The first 50 snapshots were grouped into ten five-frame blocks, and the remaining single snapshot was excluded from the block level statistical comparison. Block averaged total energies of IA and the corresponding reference ligand for each target were compared using Welch’s *t*-test, with *p* < 0.05 considered statistically significant.

### 4.5. Protein–Protein Interaction (PPI) Analysis

Protein–protein interaction analysis was performed for the proteins INSR, IRS1, AKT, AS160, SIRT1, SIRT3, SIRT6, NF-κB, NRF2 and PPARα using the STRING database [64]. Nodes and edges represent proteins and known/predicted interactions respectively. Edge colors were represented as in the STRING database legend.

### 4.6. Neuronal Differentiation and Characterization of Bone Marrow-Derived Mesenchymal Stem Cells

Bone marrow-derived mesenchymal stem cells (BM-MSCs) were cultured in Low Glucose Dulbecco’s Modified Eagle Medium (DMEM) (Capricorn Scientific GmbH, Ebsdorfergrund, Germany) with 10% Fetal Bovine Serum (FBS) (Capricorn Scientific GmbH, Ebsdorfergrund, Germany) and 1% Penicillin/Streptomycin (P/S) (Gibco, Waltham, MA, USA in a cell culture incubator with 5% CO_2_ and 95% humidity. Neuronal differentiation was conducted as in the study by Karakas et al. [35]. Basically, when cells reached 70–80% confluency, neurobasal medium (Gibco, Waltham, MA, USA) including 20 ng/mL EGF (Epidermal Growth Factor) (BioLegend, San Diego, CA, USA) 40 ng/mL bFGF (basic Fibroblast Growth Factor) (BioLegend, San Diego, CA, USA), 10 ng/mL FGF-8a (Fibroblast Growth Factor 8) (BioLegend, San Diego, CA, USA), 10 ng/mL BDNF (Brain Derived Neurotrophic Factor) (BioLegend, San Diego, CA, USA), 40 ng/mL b-NGF (Nerve Growth Factor) (BioLegend, San Diego, CA, USA), 0.125 mg/mL dbcAMP (dibutyryl cyclic AMP) (Adooq Bioscience, Irvine, CA, USA), 0.5 mM IBMX (3-isobutyl-1-methylxanthine) (Sigma-Aldrich, St. Louis, MO, USA), 2 mM L-glutamine (Gibco, Waltham, MA, USA), and 1X B27 (Gibco, Waltham, MA, USA) was introduced, and differentiation had started. Neuronal induction medium was changed every 48 h.

The differentiation process was evaluated by immunofluorescent staining of differentiated cells with the neuronal markers NeuN and synaptophysin on day 6 of differentiation [38,39]. Marker-positive cells were quantified using a Fiji/ImageJ Cell Counter.(version 1.54t) DAPI-positive nuclei were considered the total cell population, and Neu or synaptophysin-positive cells were expressed as a percentage of the total DAPI-positive cell population.

### 4.7. Incensole Acetate Exposure and MTT Analysis

In a 96-well cell culture plate, 1 × 10^4^ cells/wells were seeded and differentiated. On day 4 of the differentiation, 10 µM amyloid beta (Aβ) (Adooq Bioscience, Irvine, CA, USA) was applied to establish the experimental AD model as our previous studies [38,39]. On the following day, 10 μM, 20 μM, 40 μM, 60 μM, 80 μM, 100 μM and 125 μM incensole acetate (MedChemExpress, Monmouth Junction, NJ, USA) dissolved in DMSO (Santa Cruz Biotechnology, Dallas, TX, USA) was added to culture medium, and 24 h incubation was conducted. The next day, 10 µl of MTT solution (5 mg/mL) (CliniSciences, Nanterre, France) was applied and incubated at 37 °C for 3 h. Formed formazan crystals were dissolved with 100 µl DMSO, and after 15 min at room temperature, absorbance was read in a spectrophotometer (AMR-100, Allsheng, Hangzhou, Zhejiang, China). The MTT assay was performed in three independent experiments, with 3–5 technical replicate wells per condition in each experiment.

### 4.8. Experimental Groups and Treatment Regimen

For subsequent molecular analyses, differentiated cells were divided into four experimental groups: Control, Vehicle, Aβ, and Aβ+IA. Aβ1–42 was prepared in NH_4_OH (Merck KGaA, Darmstadt, Germany), whereas IA was prepared in DMSO. The Control group consisted of differentiated cells without Aβ1–42, IA, or vehicle exposure. The Vehicle group received the corresponding volume of NH_4_OH on day 4 of neuronal differentiation and the corresponding volume of DMSO on day 5. In the Aβ group, cells were exposed to 10 μM Aβ1–42 on day 4 and received the corresponding volume of DMSO without IA on day 5. In the Aβ+IA group, cells were exposed to 10 μM Aβ1–42 on day 4 and subsequently treated with 40 μM IA on day 5. Following 24 h of IA exposure, cells were collected on day 6 for RNA and protein isolation or processed for immunofluorescence analysis, as appropriate.

### 4.9. RNA Isolation, cDNA Synthesis and RT-qPCR Analysis

Cells were seeded in a 6-well cell culture plate as 15 × 10^4^ cells/wells and differentiated with neuronal induction medium. A 10 µM Aβ and 40 µM IA treatment was conducted on the 4th and 5th day of differentiation respectively. On day 6, cells were detached, and the RNA isolation process was started with Trizol reagent (Ambion, Waltham, MA, USA, followed by RNA isolation kit (Ambion, Waltham, MA, USA) according to the manufacturer’s protocol. A total of 100 ng total RNA sample was converted to cDNA by AB Clonal High-Capacity cDNA Reverse Transcriptase kit (Applied Biosystems, Waltham, MA, USA).

RT-qPCR analyses were carried out in a Light Cycler 480 II (Roche Diagnostics GmbH, Mannheim, Germany) withSYBR Green master mix and ACTB was used as the housekeeping control. Relative mRNA expression levels were calculated with the 2^−ΔΔCt^ method in GeneGlobe Data Analysis Center (https://geneglobe.qiagen.com/, accessed on 7 July 2024) and represented as fold change according to the control group [65]. The primer sequences are depicted in Table 3. RT-qPCR analyses were performed using RNA obtained from three independent isolations, and each PCR measurement was conducted in at least three technical replicate wells.

### 4.10. Total Protein Isolation and Western Blot

A total of 40 µM IA was applied to the AB-exposed cells on day 5 of differentiation, and the next day, cells were collected and homogenized with lysis buffer (150 mM NaCl (Merck KGaA, Darmstadt, Germany), 50 mM Tris (Sigma-Aldrich, St. Louis, MO, USA) (pH:8), 1% Triton-X-100 (Sigma-Aldrich, St. Louis, MO, USA)) with 1% Protease/Phosphatase inhibitor cocktail (Cell Signaling Technology, Danvers, MA, USA). A total of 40–80 µg of total protein was denatured and ran within 4–12% Bis-Tris gel (Invitrogen, Waltham, MA, USA) under an electrical field. Next, proteins were transferred onto a PVDF membrane (Invitrogen, Waltham, MA, USA) and blocked with 5% skimmed milk (BioShop Canada Inc., Burlington, ON, Canada) or 5% BSA (Sigma-Aldrich, St. Louis, MO, USA). After blocking, process membranes were incubated with appropriate primary antibodies (pINSR 1:500, STJ90816, St John’s Laboratory Ltd., London, UK; tINSR 1:500, sc-81465, Santa Cruz Biotechnology, Dallas, TX, USA; pAKT 1:1000, 4051s, Cell Signaling Technology, Danvers, MA, USA; tAKT 1:1000, 9277s, Cell Signaling Technology, Danvers, MA, USA; AS160 1:500, E-AB-13723, Elabscience, Houston, TX, USA; SIRT1 1:1000, 9475s, Cell Signaling Technology, Danvers, MA, USA; SIRT3 1:1000, 5490s, Cell Signaling Technology, Danvers, MA, USA; SIRT6 1:1000, 12486s, Cell Signaling Technology, Danvers, MA, USA; NFκB 1:1000, 8242s, Cell Signaling Technology, Danvers, MA, USA; NRF2 1:1000, 8882s, Cell Signaling Technology, Danvers, MA, USA; Beta actin 1:1000, sc-47778, Santa Cruz Biotechnology, Dallas, TX, USA). After the incubation, membranes were washed with TBST and incubated with HRP conjugated secondary antibodies (goat anti mouse 1:2500 405306 Biolegend, San Diego, CA, USA; Donkey anti rabbit 1:2500 406401 Biolegend, San Diego, CA, USA). Chemiluminescent images were obtained with iBright FL1000 Imaging System (Thermo Fisher Scientific, Waltham, MA, USA) and analyzed using Image J software (version 1.54i) (NIH, NIH, Bethesda, MD, USA). Phosphorylated proteins were normalized according to total proteins, while total proteins were normalized according to beta actin levels [66]. Western blot analyses were performed in three independent experiments.

### 4.11. Immunofluorescence Staining

Cells were seeded in a 24-well plate as 5 × 10^4^ cells/well, and the AD model was constituted. Following IA treatment, cells were fixed with 4% paraformaldehyde (PFA) at room temperature for 15 min. Afterwards, blocking was carried out by incubating cells within PBS-containing 1% BSA. Cells were incubated with primary antibody PPARα (1:50, Santa Cruz Biotechnology, Dallas, TX, USA) at 4 °C overnight. The following day, cells were washed with PBS and incubated with fluorescent-tagged secondary antibody (1:250, Alexa Flour 488 goat anti mouse, A-11001, Thermo Fisher Scientific, Waltham, MA, USA) at room temperature for 1 h, and nuclei were stained with DAPI. Immunofluorescent staining was determined by an EVOS FL microscope (Life Technologies Corporation, Bothell, WA, USA). Fluorescent intensity was measured by Fiji, ImageJ 1.54i software and analyzed with the modified H-SCORE method by having numerical scores 0 to 300 [67].

### 4.12. Statistical Analyses

All statistical analyses were conducted by using GraphPad Prism Version 5.01 (GraphPad Software Inc., San Diego, CA, USA). To determine the significance between groups, one-way ANOVA, followed by Tukey’s test, was used. All values were represented as mean ± standard deviation (SD), and *** *p* < 0.001, ** *p* < 0.01 and * *p* < 0.05 were accepted as statistically significant.

## 5. Conclusions

In conclusion, the present findings indicate that IA treatment is associated with changes in insulin-signaling-related proteins, sirtuin expression, inflammatory and oxidative-stress markers, and PPARα in an Aβ1–42-induced cellular AD model. Computational analyses further demonstrated predicted interactions between IA and SIRT1, SIRT3, and SIRT6, with particularly favorable dynamic binding energetics toward SIRT6. However, these computational and expression-based findings do not establish direct sirtuin activation or demonstrate that the effects of IA on insulin signaling are mediated through sirtuins. In addition, IA treatment was associated with reduced detectable Aβ levels, although the mechanism underlying this reduction could not be determined in the present study. Targeted experiments using sirtuin-specific inhibition or knockdown, together with in vivo studies, will be required to establish the mechanistic contribution of individual sirtuins and further evaluate the therapeutic potential of IA in AD.

## Figures and Tables

**Table 1:** Predicted physicochemical, pharmacokinetic, metabolic, and toxicity properties of incensole acetate, reference ligands, and aflatoxin B1 as a positive toxic/carcinogenic control.

Parameters by SwissADME	Incensole Acetate	4TQ	BBI	8L9	Aflatoxin B1 (Positive Control)
**Physicochemical properties**					
**MW (g/mol)**	348.52	493.48	645.31	612.27	312.06
**Aromatic atoms**	0	23	15	18	10
**Heavy atoms**	25	36	31	34	23
**Molar refractivity**	105.14	136.56	144.33	130.27	79.48
**H-bond acceptors**	3	7	4	7	6
**H-bond donors**	0	1	0	3	0
**TPSA (Å^2^)**	35.53	74.50	42.68	146.40	74.97
**Rotatable bonds**	3	6	11	7	1
**Lipophilicity**					
**Consensus Log Po/w**	4.62	4.51	6.49	4.03	1.70
**Water solubility**					
**ESOL Log S**	Moderately soluble	Poorly soluble	Poorly soluble	Poorly soluble	Soluble
**Drug likeness**					
**Lipinski violations**	0	0	2	1	0
**Medicinal Chemistry**					
**Synthetic accessibility**	6.07	4.37	4.00	3.40	4.29
**Pharmacokinetics**					
**GI absorption**	High	High	Low	Low	High
**BBB permeant**	Yes	No	No	No	Yes
**Pgp substrate**	No	Yes	Yes	No	No
**CYP1A2 inhibitor**	No	No	No	No	No
**CYP2C19 inhibitor**	No	Yes	No	No	Yes
**CYP2C9 inhibitor**	Yes	Yes	No	Yes	No
**CYP2D6 inhibitor**	No	Yes	No	No	Yes
**CYP3A4 inhibitor**	Yes	Yes	No	No	Yes
**Outputs of toxicity classification endpoints by ADMETlab 3.0**					
**H-HT (Hepatoxicity)**	0.729	0.877	0.719	0.907	0.881
**DILI (Drug Induced Liver Injury)**	0.766	0.984	0.217	1.0	0.995
**ROA (Rat Oral Acute)**	0.276	0.655	0.601	0.291	0.886
**Carcinogenicity**	0.458	0.434	0.478	0.155	0.987
**Toxicophore Rules**					
**Acute Toxicity**	0	0	0	0	0
**NonGenotoxic_Carcinogenicity**	0	0	0	0	0
**Genotoxic_Carcinogenicity_Mutagenicity**	0	1	0	1	3

**Table 2:** Interacting residues of IA and reference activators and their predicted binding affinities in PDB ID: 4ZZJ (SIRT1), 5H4D (SIRT3) and 5Y2F (SIRT6).

Target Proteins	Compound (Ligand)	Binding Affinity (kcal/mol)	Distance (A)	Interaction Residue Numbers	
				Hydrogen Bonds	Hydrophobic İnteractions
**SIRT1 (4ZZJ)**	IA	−3.270	3.36–3.66		LEU24, ILE41, ILE45, GLU48
	4TQ	−6.246	2.80–3.97	ASN44	THR37, GLN40, ILE41, ILE45
**SIRT3 (5H4D)**	IA	−5.944	3.39–4.18	ARG158	PHE157, PHE180, GLN228, ILE230, HIS248, ILE291
	BBI	−6.960	2.85–5.0	ARG158	ALA146, PHE157, ARG158, PRO176, GLU177, PHE180, ILE230, HIS248, PHE294
**SIRT6 (5Y2F)**	IA	−5.244	3.34–3.87	PHE84	PRO60, VAL68, PRO78, PHE80
	8L9	−7.572	2.68–5.02	ASN2, GLU72	ASN2, ILE59, VAL68, PRO78, PHE80, PHE84

**Table 3:** Primer sequences of the target genes analyzed in this study.

Gene	Primer Sequences
INSR-F	GAACTACAGCGTGCGAATCC
INSR-R	GAAAGGACAAAGATGAGGGGG
IRS-F	ACTGACAACGAGAGCCATGA
IRS-R	TGGCTTCCACCCATTCTTCT
AKT-F	GGGTGCATTTGAGAGAAGCC
AKT-R	CACACCCGGAGAACAAACTG
AS160-F	CCTGTTATGAACCAGGCTGC
AS160-R	TCCTGCAACTAGACGATCCC
ACTB-F	CATGTACGTTGCTATCCAGGC
ACTB-R	CTCCTTAATGTCACGCACGAT

## Data Availability

The original contributions presented in this study are included in the article/Supplementary Materials. Further inquiries can be directed to the corresponding author.

## Supplementary Materials

The following supporting information can be downloaded at: https://www.mdpi.com/article/10.3390/ph19101573/s1, Figure S1: Two-dimensional interaction diagram of the best-ranked docked pose of EX-527 in the investigated SIRT1 inhibitor-binding region. EX-527 was included as a positive-control assessment of the docking protocol. Table S1. MM-PBSA energy decomposition of IA and the corresponding reference ligand complexes. Values represent mean ± SD across 51 analyzed snapshots and are expressed in kcal/mol. Entropic contributions were not available in the exported MM-PBSA output and are therefore not included.

## References

[1] Andrade-Guerrero J., Santiago-Balmaseda A., Jeronimo-Aguilar P., Vargas-Rodríguez I., Cadena-Suárez A.R., Sánchez-Garibay C., Pozo-Molina G., Méndez-Catalá C.F., Cardenas-Aguayo M.-C., Diaz-Cintra S. (2023). Alzheimer’s Disease: An Updated Overview of Its Genetics. Int. J. Mol. Sci..

[2] Long J.M., Holtzman D.M. (2019). Alzheimer Disease: An Update on Pathobiology and Treatment Strategies. Cell.

[3] García-Morales V., González-Acedo A., Melguizo-Rodríguez L., Pardo-Moreno T., Costela-Ruiz V.J., Montiel-Troya M., Ramos-Rodríguez J.J. (2021). Current Understanding of the Physiopathology, Diagnosis and Therapeutic Approach to Alzheimer’s Disease. Biomedicines.

[4] Haeusler R.A., McGraw T.E., Accili D. (2018). Biochemical and Cellular Properties of Insulin Receptor Signalling. Nat. Rev. Mol. Cell Biol..

[5] Le T.K.C., Dao X.D., Nguyen D.V., Luu D.H., Bui T.M.H., Le T.H., Nguyen H.T., Le T.N., Hosaka T., Nguyen T.T.T. (2023). Insulin Signaling and Its Application. Front. Endocrinol..

[6] Yunn N.O., Kim J., Ryu S.H., Cho Y. (2023). A Stepwise Activation Model for the Insulin Receptor. Exp. Mol. Med..

[7] Kulas J.A., Weigel T.K., Ferris H.A. (2020). Insulin Resistance and Impaired Lipid Metabolism as a Potential Link between Diabetes and Alzheimer’s Disease. Drug Dev. Res..

[8] Shieh J.C.C., Huang P.T., Lin Y.F. (2020). Alzheimer’s Disease and Diabetes: Insulin Signaling as the Bridge Linking Two Pathologies. Mol. Neurobiol..

[9] Arnold S.E., Arvanitakis Z., Macauley-Rambach S.L., Koenig A.M., Wang H.Y., Ahima R.S., Craft S., Gandy S., Buettner C., Stoeckel L.E. (2018). Brain Insulin Resistance in Type 2 Diabetes and Alzheimer Disease: Concepts and Conundrums. Nat. Rev. Neurol..

[10] Gali C.C., Fanaee-Danesh E., Zandl-Lang M., Albrecher N.M., Tam-Amersdorfer C., Stracke A., Sachdev V., Reichmann F., Sun Y., Avdili A. (2019). Amyloid-Beta Impairs Insulin Signaling by Accelerating Autophagy-Lysosomal Degradation of LRP-1 and IR-β in Blood-Brain Barrier Endothelial Cells In Vitro and in 3xTg-AD Mice. Mol. Cell. Neurosci..

[11] Nguyen T.T., Ta Q.T.H., Nguyen T.T.D., Le T.T., Vo V.G. (2020). Role of Insulin Resistance in the Alzheimer’s Disease Progression. Neurochem. Res..

[12] Kellar D., Craft S. (2020). Brain Insulin Resistance in Alzheimer’s Disease and Related Disorders: Mechanisms and Therapeutic Approaches. Lancet Neurol..

[13] Arvanitakis Z., Wang H.Y., Capuano A.W., Khan A., Taïb B., Anokye-Danso F., Schneider J.A., Bennett D.A., Ahima R.S., Arnold S.E. (2020). Brain Insulin Signaling, Alzheimer Disease Pathology, and Cognitive Function. Ann. Neurol..

[14] Akhtar A., Sah S.P. (2020). Insulin Signaling Pathway and Related Molecules: Role in Neurodegeneration and Alzheimer’s Disease. Neurochem. Int..

[15] Gonçalves R.A., Wijesekara N., Fraser P.E., De Felice F.G. (2019). The Link between Tau and Insulin Signaling: Implications for Alzheimer’s Disease and Other Tauopathies. Front. Cell. Neurosci..

[16] Ohyagi Y., Takei S.I. (2020). Insulin Signaling as a Therapeutic Target in Alzheimer’s Disease: Efficacy of Apomorphine. Neurol. Clin. Neurosci..

[17] Kanwal A., Dsouza L.A. (2019). Sirtuins and Diabetes: Optimizing the Sweetness in the Blood. Transl. Med. Commun..

[18] Kitada M., Ogura Y., Monno I., Koya D. (2019). Sirtuins and Type 2 Diabetes: Role in Inflammation, Oxidative Stress, and Mitochondrial Function. Front. Endocrinol..

[19] Wang J., Zhou F., Xiong C.E., Wang G.P., Chen L.W., Zhang Y.T., Qi S.G., Wang Z.H., Mei C., Xu Y.J. (2023). Serum Sirtuin1: A Potential Blood Biomarker for Early Diagnosis of Alzheimer’s Disease. Aging.

[20] Lee J., Kim Y., Liu T., Hwang Y.J., Hyeon S.J., Im H., Lee K., Alvarez V.E., McKee A.C., Um S.J. (2018). SIRT3 Deregulation Is Linked to Mitochondrial Dysfunction in Alzheimer’s Disease. Aging Cell.

[21] Pradhan R., Singh A.K., Kumar P., Bajpai S., Pathak M., Chatterjee P., Dwivedi S., Dey A.B., Dey S. (2022). Blood Circulatory Level of Seven Sirtuins in Alzheimer’s Disease: Potent Biomarker Based on Translational Research. Mol. Neurobiol..

[22] Al-Harrasi A., Csuk R., Khan A., Hussain J. (2019). Distribution of the Anti-Inflammatory and Anti-Depressant Compounds: Incensole and Incensole Acetate in Genus Boswellia. Phytochemistry.

[23] Doaee P., Rajaei Z., Roghani M., Alaei H., Kamalinejad M. (2019). Effects of Boswellia serrata Resin Extract on Motor Dysfunction and Brain Oxidative Stress in an Experimental Model of Parkinson’s Disease. Avicenna J. Phytomed..

[24] Moussaieff A., Mechoulam R. (2009). Boswellia Resin: From Religious Ceremonies to Medical Uses; A Review of In Vitro, In Vivo and Clinical Trials. J. Pharm. Pharmacol..

[25] Yassin N.A.Z., El-Shenawy S.M.A., Mahdy K.A., Gouda N.A.M., Marrie A.E.-F.H., Farrag A.R.H., Ibrahim B.M.M. (2013). Effect of Boswellia serrata on Alzheimer’s Disease Induced in Rats. J. Arab Soc. Med. Res..

[26] El-Magd M.A., Khalifa S.F., Faisal F.A., Badawy A.A., El-Shetry E.S., Dawood L.M., Alruwaili M.M., Alrawaili H.A., Risha E.F., El-Taweel F.M. (2018). Incensole Acetate Prevents Beta-Amyloid-Induced Neurotoxicity in Human Olfactory Bulb Neural Stem Cells. Biomed. Pharmacother..

[27] Moussaieff A., Shohami E., Kashman Y., Fride E., Schmitz M.L., Renner F., Fiebich B.L., Munoz E., Ben-Neriah Y., Mechoulam R. (2007). Incensole Acetate, a Novel Anti-Inflammatory Compound Isolated from Boswellia Resin, Inhibits Nuclear Factor-κB Activation. Mol. Pharmacol..

[28] Ittner L.M., Götz J. (2011). Amyloid-β and Tau—A Toxic Pas de Deux in Alzheimer’s Disease. Nat. Rev. Neurosci..

[29] Ramasubbu K., Devi Rajeswari V. (2023). Impairment of Insulin Signaling Pathway PI3K/Akt/mTOR and Insulin Resistance Induced AGEs on Diabetes Mellitus and Neurodegenerative Diseases: A Perspective Review. Mol. Cell. Biochem..

[30] Michailidis M., Moraitou D., Tata D.A., Kalinderi K., Papamitsou T., Papaliagkas V. (2022). Alzheimer’s Disease as Type 3 Diabetes: Common Pathophysiological Mechanisms between Alzheimer’s Disease and Type 2 Diabetes. Int. J. Mol. Sci..

[31] Gawade A., Polshettiwar S., Hingalajia H., Prajapati B.G., Singh A. (2024). Pharmacokinetics and Pharmacodynamics of Various Novel Formulations Targeting Alzheimer’s Disease. Alzheimer’s Disease and Advanced Drug Delivery Strategies.

[32] Agu P.C., Afiukwa C.A., Orji O.U., Ezeh E.M., Ofoke I.H., Ogbu C.O., Ugwuja E.I., Aja P.M. (2023). Molecular Docking as a Tool for the Discovery of Molecular Targets of Nutraceuticals in Diseases Management. Sci. Rep..

[33] Durrant J.D., McCammon J.A. (2011). Molecular Dynamics Simulations and Drug Discovery. BMC Biol..

[34] Genheden S., Ryde U. (2015). The MM/PBSA and MM/GBSA Methods to Estimate Ligand-Binding Affinities. Expert Opin. Drug Discov..

[35] Karakaş N., Bay S., Türkel N., Öztunç N., Öncül M., Bilgen H., Shah K., Şahin F., Öztürk G. (2020). Neurons from Human Mesenchymal Stem Cells Display Both Spontaneous and Stimuli Responsive Activity. PLoS ONE.

[36] Song C.G., Zhang Y.Z., Wu H.N., Cao X.L., Guo C.J., Li Y.Q., Zheng M.H., Han H. (2018). Stem Cells: A Promising Candidate to Treat Neurological Disorders. Neural Regen. Res..

[37] Carolindah M.N., Rosli R., Adam A., Nordin N. (2013). An Overview of In Vitro Research Models for Alzheimer’s Disease (AD). Regen. Res..

[38] Ercin N., Besli N., Johnson B.S., Cakmak R.K., Beker M., Beker M.C., Celik U. (2025). Investigation of the Effects of Acacetin on Autophagy Pathway and Exosome Release in Amyloid Beta Peptide-Induced Toxicity Models. Mol. Neurobiol..

[39] Sarikamis Johnson B., Ercin N., Kalkan Cakmak R., Besli N., Beker M., Beker M.C., Celik U. (2025). Exploring the Effects of Squalene in the PERK/ATF4/EIF2α/CHOP Signalling Pathway in an In Vitro Alzheimer Disease Model and In Silico Approach. Metab. Brain Dis..

[40] Gabbouj S., Ryhänen S., Marttinen M., Wittrahm R., Takalo M., Kemppainen S., Martiskainen H., Tanila H., Haapasalo A., Hiltunen M. (2019). Altered Insulin Signaling in Alzheimer’s Disease Brain—Special Emphasis on PI3K-Akt Pathway. Front. Neurosci..

[41] Gupta S., Yadav K., Mantri S.S., Singhal N.K., Ganesh S., Sandhir R. (2018). Evidence for Compromised Insulin Signaling and Neuronal Vulnerability in Experimental Model of Sporadic Alzheimer’s Disease. Mol. Neurobiol..

[42] Liu Y., Liu F., Grundke-Iqbal I., Iqbal K., Gong C.-X. (2011). Deficient Brain Insulin Signalling Pathway in Alzheimer’s Disease and Diabetes. J. Pathol..

[43] Liu Y., Beyer A., Aebersold R. (2016). On the Dependency of Cellular Protein Levels on mRNA Abundance. Cell.

[44] Hölscher C. (2019). Insulin Signaling Impairment in the Brain as a Risk Factor in Alzheimer’s Disease. Front. Aging Neurosci..

[45] Ma Q.L., Yang F., Rosario E.R., Ubeda O.J., Beech W., Gant D.J., Chen P.P., Hudspeth B., Chen C., Zhao Y. (2009). β-Amyloid Oligomers Induce Phosphorylation of Tau and Inactivation of Insulin Receptor Substrate via c-Jun N-Terminal Kinase Signaling: Suppression by Omega-3 Fatty Acids and Curcumin. J. Neurosci..

[46] Das T.K., Chakrabarti S.K., Zulkipli I.N., Abdul Hamid M.R.W. (2019). Curcumin Ameliorates the Impaired Insulin Signaling Involved in the Pathogenesis of Alzheimer’s Disease in Rats. J. Alzheimer’s Dis. Rep..

[47] Lee H.-K., Kumar P., Fu Q., Rosen K.M., Querfurth H.W. (2009). The Insulin/Akt Signaling Pathway Is Targeted by Intracellular β-Amyloid. Mol. Biol. Cell.

[48] Manning B.D., Toker A. (2017). AKT/PKB Signaling: Navigating the Network. Cell.

[49] De Meyts P. (2000). The Insulin Receptor and Its Signal Transduction Network. Endotext.

[50] Liang F., Kume S., Koya D. (2009). SIRT1 and Insulin Resistance. Nat. Rev. Endocrinol..

[51] Li X., Li Y., Hao Q., Jin J., Wang Y. (2024). Metabolic Mechanisms Orchestrated by Sirtuin Family to Modulate Inflammatory Responses. Front. Immunol..

[52] Sun E., Motolani A., Campos L., Lu T. (2022). The Pivotal Role of NF-κB in the Pathogenesis and Therapeutics of Alzheimer’s Disease. Int. J. Mol. Sci..

[53] Davies D.A., Adlimoghaddam A., Albensi B.C. (2021). Role of Nrf2 in Synaptic Plasticity and Memory in Alzheimer’s Disease. Cells.

[54] Ramsey C.P., Glass C.A., Montgomery M.B., Lindl K.A., Ritson G.P., Chia L.A., Hamilton R.L., Chu C.T., Jordan-Sciutto K.L. (2007). Expression of Nrf2 in Neurodegenerative Diseases. J. Neuropathol. Exp. Neurol..

[55] Assaf N., El-Shamarka M.E., Salem N.A., Khadrawy Y.A., El Sayed N.S. (2020). Neuroprotective Effect of PPAR Alpha and Gamma Agonists in a Mouse Model of Amyloidogenesis through Modulation of the Wnt/Beta-Catenin Pathway via Targeting Alpha- and Beta-Secretases. Prog. Neuro-Psychopharmacol. Biol. Psychiatry.

[56] De La Monte S.M., Wands J.R. (2006). Molecular Indices of Oxidative Stress and Mitochondrial Dysfunction Occur Early and Often Progress with Severity of Alzheimer’s Disease. J. Alzheimer’s Dis..

[57] Shao Y., Chen J., Dong L.J., He X., Cheng R., Zhou K., Liu J., Qiu F., Li X.R., Ma J.X. (2019). A Protective Effect of PPARα in Endothelial Progenitor Cells through Regulating Metabolism. Diabetes.

[58] Daina A., Michielin O., Zoete V. (2017). SwissADME: A Free Web Tool to Evaluate Pharmacokinetics, Drug-Likeness and Medicinal Chemistry Friendliness of Small Molecules. Sci. Rep..

[59] Fu L., Shi S., Yi J., Wang N., He Y., Wu Z., Peng J., Deng Y., Wang W., Wu C. (2024). ADMETlab 3.0: An Updated Comprehensive Online ADMET Prediction Platform Enhanced with Broader Coverage, Improved Performance, API Functionality and Decision Support. Nucleic Acids Res..

[60] Besli N., Ercin N., Kalkan-Cakmak R., Sarikamis-Johnson B., Beker M., Celik U. (2025). Discovering the Natural Source-Derived Antihypertensive Compounds Aspiring Current Therapeutic Targets by Computer-Based Drug Design. Biochem. Biophys. Res. Commun..

[61] Cakmak R.K., Besli N., Ercin N., Celik U. (2026). AI-Powered Literature Mining Reveals the Therapeutic Significance of GLP-1 Receptor: Simulation of Natural Agonist Candidates Based on Molecular Dynamics. Comput. Biol. Chem..

[62] Van Der Spoel D., Lindahl E., Hess B., Groenhof G., Mark A.E., Berendsen H.J.C. (2005). GROMACS: Fast, Flexible, and Free. J. Comput. Chem..

[63] Valdés-Tresanco M.S., Valdés-Tresanco M.E., Valiente P.A., Moreno E. (2021). gmx_MMPBSA: A New Tool to Perform End-State Free Energy Calculations with GROMACS. J. Chem. Theory Comput..

[64] Szklarczyk D., Kirsch R., Koutrouli M., Nastou K., Mehryary F., Hachilif R., Gable A.L., Fang T., Doncheva N.T., Pyysalo S. (2023). The STRING Database in 2023: Protein-Protein Association Networks and Functional Enrichment Analyses for Any Sequenced Genome of Interest. Nucleic Acids Res..

[65] Kilic E., Kilic Ü., Bacigaluppi M., Guo Z., Abdallah N.B., Wolfer D.P., Reiter R.J., Hermann D.M., Bassetti C.L. (2008). Delayed Melatonin Administration Promotes Neuronal Survival, Neurogenesis and Motor Recovery, and Attenuates Hyperactivity and Anxiety after Mild Focal Cerebral Ischemia in Mice. J. Pineal Res..

[66] Beker M., Gunay N., Sarikamis B., Cakmak R.K., Ercin N., Altintas M.O., Altunay S., Beker M.C., Ak D.S., Kilic U. (2023). Dual Action of Exosomes Derived from In Vitro Aβ Toxicity Model: The Role of Age for Pathological Response. Arch. Gerontol. Geriatr..

[67] Besli N., Ercin N., Carmena-Bargueño M., Sarikamis B., Kalkan Cakmak R., Yenmis G., Pérez-Sánchez H., Beker M., Kilic U. (2024). Research into How Carvacrol and Metformin Affect Several Human Proteins in a Hyperglycemic Condition: A Comparative Study In Silico and In Vitro. Arch. Biochem. Biophys..

