# Supplementary material for: Incensole Acetate Regulates the Sirtuin–Insulin Signaling Axis in an Experimental Alzheimer’s Disease Model
Source: Pharmaceuticals (Basel). 2026 Oct 3;19(10):1573. doi: 10.3390/ph19101573 (PMC13708164; doi:10.3390/ph19101573)
Supplement: Supplementary file 1 [file pharmaceuticals-19-01573-s001.zip › pharmaceuticals-4540454-supplementary.pdf]

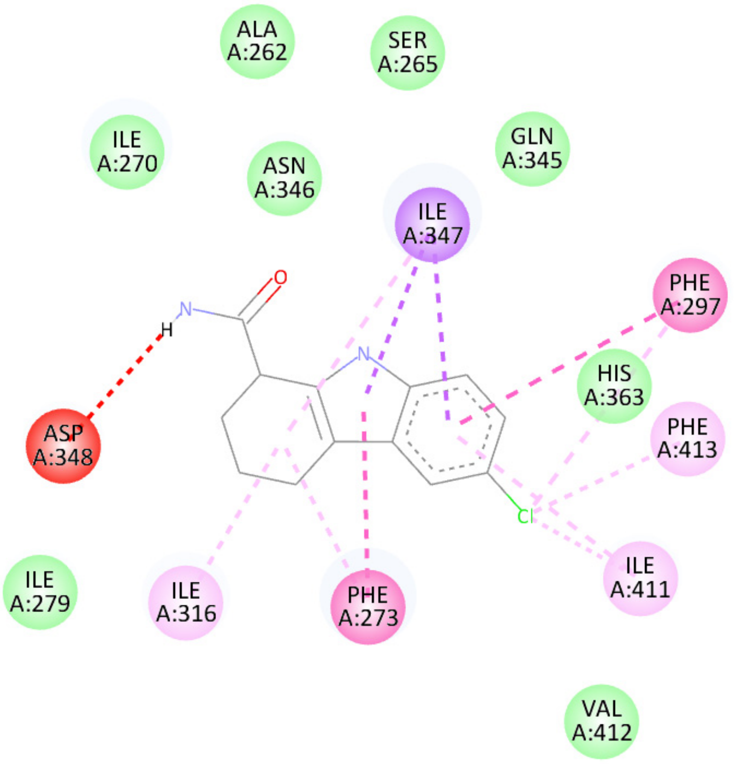

Supplementary Figure S1: Two-dimensional interaction diagram of the best-ranked docked pose of EX-527 in the investigated SIRT1 inhibitor-binding region. EX-527 was included as a positive-control assessment of the docking protocol.

Supplementary Table S1. MM-PBSA energy decomposition of IA and the corresponding reference ligand complexes. Values represent mean ± SD across 51 analyzed snapshots and are expressed in kcal/mol. Entropic contributions were not available in the exported MM-PBSA output and are therefore not included.

**Complex**	**van der Waals**	**Electrostatic**	**Polar solvation**	**Non-polar solvation**	**Total MM-PBSA**
**SIRT1-4TQ**	−44.25 ± 7.94	−1.31 ± 1.81	11.98 ± 4.43	−3.71 ± 0.42	**−37.29 ± 5.49**
**SIRT1-IA**	−46.63 ± 2.35	−1.10 ± 1.80	16.92 ± 3.80	−4.36 ± 0.10	**−35.16 ± 3.32**
**SIRT3-BBI**	−62.72 ± 9.16	−3.05 ± 0.96	18.28 ± 2.64	−4.63 ± 0.36	**−52.12 ± 8.61**
**SIRT3-IA**	−41.06 ± 4.19	−1.86 ± 1.56	16.34 ± 3.39	−3.66 ± 0.14	**−30.24 ± 3.63**
**SIRT6-8L9**	−31.70 ± 6.00	−53.18 ± 27.92	67.15 ± 25.38	−3.33 ± 0.42	**−21.06 ± 6.16**
**SIRT6-IA**	−50.96 ± 3.83	−1.08 ± 1.09	17.61 ± 2.35	−3.83 ± 0.10	**−38.26 ± 3.90**
